# Evaluation of antiplasmodial activity in silico and in vitro of *N*-acylhydrazone derivatives

**DOI:** 10.1186/s13065-022-00843-9

**Published:** 2022-07-09

**Authors:** Fernanda A. Oliveira, Ana Claudia S. Pinto, Caique L. Duarte, Alex G. Taranto, Eder Lorenzato Junior, Cleydson Finotti Cordeiro, Diogo T. Carvalho, Fernando P. Varotti, Amanda L. Fonseca

**Affiliations:** 1 Núcleo de Pesquisa Em Química Biológica (NQBio), Universidade Federal de São João Del Rei, Campus Centro Oeste, Divinópolis, MG 35501-296 Brazil; 2 Laboratório de Pesquisa Em Química Farmacêutica, Universidade Federal de Alfenas, Campus Alfenas, Alfenas, MG 37130-001 Brazil

**Keywords:** Malaria, Bioinformatics, Molecular modeling

## Abstract

*N*-acylhydrazones are considered privileged structures in medicinal chemistry, being part of antimicrobial compounds (for example). In this study we show the activity of *N*-acylhydrazone compounds, namely AH1, AH2, AH4, AH5 in in vitro tests against the chloroquine-resistant strain of *Plasmodium falciparum* (W2) and against WI26 VA-4 human cell lines. All compounds showed low cytotoxicity (LC_50_ > 100 µM). The AH5 compound was the most active against *Plasmodium falciparum*, with an IC_50_ value of 0.07 μM. AH4 and AH5 were selected among the tested compounds for molecular docking calculations to elucidate possible targets involved in their mechanism of action and the SwissADME analysis to predict their pharmacokinetic profile. The AH5 compound showed affinity for 12 targets with low selectivity, while the AH4 compound had greater affinity for only one target (3PHC). These compounds met Lipinski's standards in the ADME in silico tests, indicating good bioavailability results. These results demonstrate that these *N*-acylhydrazone compounds are good candidates for future preclinical studies against malaria.

**Graphical Abstract:**

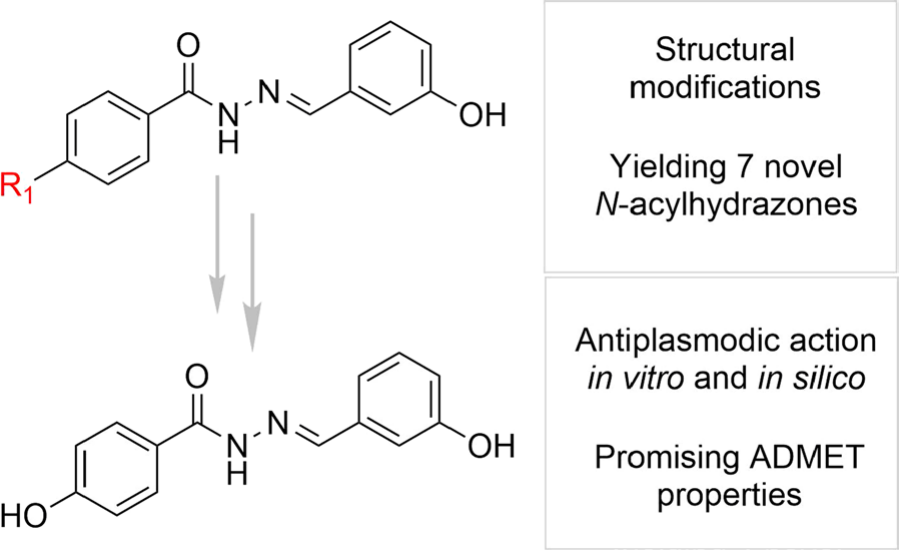

**Supplementary Information:**

The online version contains supplementary material available at 10.1186/s13065-022-00843-9.

## Introduction

Malaria is an infectious disease caused by protozoa of the genus *Plasmodium* spp., and remains one of the major public health problems worldwide [1]. A total of 241 million cases and approximately 627,000 deaths were reported in 2020. The numbers represent a 6% increase in case reports compared to 2019 numbers. Increase associated with interruption of prevention and treatment during the COVID-19 pandemic, especially in Sub-Saharan Africa [2].

Five species are capable of infecting humans: *Plasmodium falciparum*, *Plasmodium vivax, Plasmodium ovale, Plasmodium malariae* and *Plasmodium knowlesi* [3]. The *P. vivax* and *P. falciparum* species are responsible for the majority of infections, with the latter being responsible for the largest number of deaths [4]. Correct identification of the infecting species is necessary to make an adequate therapeutic decision [5].

Malaria treatment consists of drugs based on natural products or synthetic compounds. Antimalarial drugs are delimited by class according to structural backbone and apparent action [4]. The following classes stand out: endoperoxides; 4-aminoquinolines; antifolates; naphthoquinones and 8-aminoquinolines [6]. With the emergence of multi-resistant strains in 2006, the World Health Organization (WHO) recommended changes to the treatment protocol for uncomplicated malaria caused by *P. falciparum*, and for malaria caused by chloroquine-resistant *P. vivax*, namely Artemisinin-based Combination Therapy (ACTs) [7].

The resistance to artemisinin in *P. falciparum* was first described in 2009 in Southeast Asia, mainly in the Greater Mekong region, a river which goes through some countries such as Cambodia and Vietnam [8]. Subsequently, the emergence of resistance was reported in African and South Asian countries [9].

Specific point mutations were identified in the k13 gene in artemisinin-resistant strains (Kelch13, PlasmoDB ID: PF3D7_1343700) [4]. The mutation of this gene causes the k13 protein to interfere in the role of the ubiquitination machinery, leading to a greater induction of protective stress responses caused by artemisinin, giving parasites a better adaptation in this environment [4, 9, 10].

The current therapeutic arsenal for malaria is limited and the emergence of in vitro and in vivo cases of resistance for ACTs makes the search for new options for compounds that can be used in clinical practice even more necessary [1, 11].

The *N*-acylhydrazone subunit is an important chemical group in drug discovery [12], as compounds with this structure have shown several biological applications, such as antiparasitic, antimicrobial, anticonvulsant [13], antiviral [14], analgesic, anti-inflammatory [15] and antiproliferative action against tumor cells [16].

Melnyk and collaborators reported antiplasmodial activity of a library of acylhydrazones against *P. falciparum* chloroquine-resistant (W2) strain [17]. Küçükgüzel and collaborators described antiplasmodial activity of a series of acylhydrazones. The new compounds exhibited activity against chloroquine-resistant W2 and *P. falciparum* chloroquine-sensitive (3D7) strains [18].

The process of developing new drugs consists of finding molecules capable of altering the function of a specific molecular target [19] to produce safer, more effective and accessible drugs [20]. An important data in order to ensure the activity of these new compounds is to study parameters like absorption, distribution, metabolism, excretion and toxicity (ADMET) during the process of development. These parameters are important to predict whether the compound is capable of becoming a bioactive molecule, which provides important data for the synthesis of possible drugs [21].

Another tool would be molecular docking, a tool that has been highlighted for its practicality, low cost and efficiency [22]. Fitting methods use a variety of algorithms to find the best pose of a ligand at the binding site, determining the main forces for molecular recognition and its affinity against a respective molecular target. This tool can therefore be useful to research and design more active ligands [23–25].

The use of in silico approaches has become increasingly frequent in malaria research, mainly as a tool that helps to describe the mechanism of action and reaction of antimalarials [26–30]. Knowledge about these mechanisms helps to predict possible unwanted interactions with other targets, in addition to guiding research aimed at structurally altering existing drugs, against which the parasite is already resistant in order to make them effective again [11, 31].

In order to understand the possible mechanism of action involved in using *N*-acylhydrazones as antimalarial compounds, one can simulate their interaction with a series of already described potential molecular targets deposited in data banks such as the Brazilian Malaria Molecular Targets (BraMMT) [11]. BraMMT is a molecular target bank created and validated by a research group which contains 35 pharmacological targets for *P. falciparum* [11].

Data banks like this enable performing inverse virtual screening (IVS), which is a process involving docking a single small-molecule, or a set of them, against a series of targets using previously validated docking software for these targets [32]. This process allows establishing an affinity relationship between the studied compounds and a specific target, comparing the binding energy generated with the binding energy of the crystallographic ligand, whose affinity has already been experimentally verified [33].

In view of the above, we report herein the synthesis, in silico properties and in vitro antiplasmodial activity of seven *N*-acylhydrazones.

## Results and discussion

### Chemistry

**Scheme 1:**
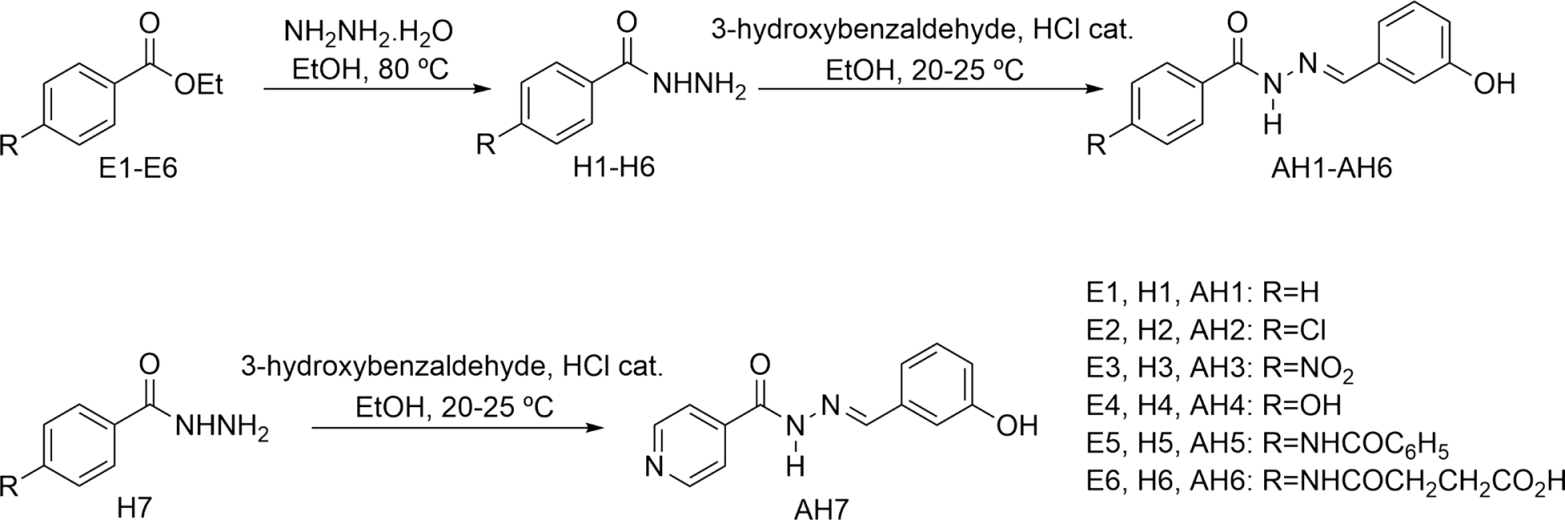
Scheme of preparation of compounds AH1-AH7

*N*-acylhydrazones (AH1-AH7) were prepared by a short synthesis route consisting in condensing the respective hydrazides H1-H7 with 3-hydroxybenzaldehyde as described previously with minor adaptations [34–38]. The hydrazides H1-H6 were obtained straightly by the hydrazinolysis of the esters E1-E6 [12–18]. The acylhydrazone HA6 is a new compound, while the others are already known substances. The overall synthesis is shown in Scheme 1.

The final products were obtained smoothly as crystalline solids in medium to high yields. The *N*-acylhydrazones were fully characterized by melting point, infrared (IR), nuclear magnetic resonance (NMR) and mass spectrometry (MS) analysis. First, the main findings in the IR spectra related to the acylhydrazones identities were the bands between 1670–1605 cm^−1^, attributed to the *N*-acylhydrazone C = N and C = O groups. The analysis of the 1H NMR spectra of AH1-AH7 showed a singlet around 8.3 ppm related to the iminic hydrogen (N = CH), which confirms the success in the condensation reaction. The same finding may be seen in 13C NMR spectra, where a signal in the range 146–148 ppm characteristic of the iminic carbon. All the other typical and expected signals for the final products were noted in the 1H and 13C NMR spectra and their identities were further confirmed by the MS analysis.

### In vitro* activity of the N-acylhydrazones*

The resistance of *Plasmodium* spp. to current drugs continues to grow and progressively limits the therapeutic arsenal available for treating patients infected with malaria. Given the above, it is necessary to discover or design new pharmacologically active compounds [39].

*N*-acylhydrazone compounds stand out in medicinal chemistry because they have a large number of successful compounds that act on several molecular targets [40, 41]. These compounds are easily obtained by synthesis, since they are usually produced by a condensation reaction between aldehydes or ketones with hydrazides, and both the starting carbonyl reagent and hydrazides may have different structural frameworks [42]. Thus, they present themselves as an economically viable strategy for developing new libraries of chemical molecules [43, 44].

A set of criteria for validation of compounds are described in the development of new antimalarial drugs. The compounds to be tested in vitro should preferably comply with specific requirements: Lethal drug concentration that reduces parasite viability by 50% (IC_50_) < 1 μM; minimum selectivity index (SI) values as 10, with the ideal being greater than 100 [11, 45].

The cytotoxicity test allows analyzing the survival rate of the cell line in the presence of the compounds. This identifies whether the compound is toxic or not to the cell line used [37]. To do so, the IC_50_ was verified. The cytotoxicity presented against human lung fibroblast cell line WI-26VA4 (ATCC CCL-95.1) was comparable to that of chloroquine and artemether.

The in vitro tests were carried out with the purpose of evaluating antiplasmodial activity of the compounds synthesized against W2 strain. Chloroquine and artemether were used as a positive control and reference (Table 1).

**Table 1:** In vitro antiplasmodial activity, cytotoxicity and selective index of *N*-acylhydrazone compounds AH1-AH7

Compounds	IC_50_ ± SD^a^ (µM)^b^*P. falciparum*	LC_50_ ± SD^a^ (µM)^c^ WI-26VA4	SI^d^
AH1	0.19 ± 0.10	100 ± 0.25	526.31
AH2	2.15 ± 0.09	100 ± 0.33	46.51
AH3	Not determined	100 ± 0.19	Not determined
AH4	0.09 ± 0.05	100 ± 0.21	1,111.11
AH5	0.07 ± 0.07	100 ± 0.23	1,428.57
AH6	Not determined	100 ± 0.17	Not determined
AH7	Not determined	100 ± 0.13	Not determined
Chloroquine	0.057 ± 0.14	100 ± 0.22	1754.38
Artemether	< 1.01	100 ± 0.18	99.01

^a^ SD: Mean and standard deviation (SD) of triplicate experiments

^b^ 50% Inhibitory concentration against a *Plasmodium falciparum* chloroquine resistant (W2) strain

^c^ 50% Cytotoxic concentration against human lung fibroblast cell line (WI-26VA4)

^d^ Selectivity index (SI) = IC_50_ (WI26VA4)/IC_50_ (*P. falciparum*)

The AH1, AH2, AH4, and AH5 compounds were active against W2 strain, and the IC_50_ determined by the traditional test ranged from 0.07 to 2.15 µM. They did not demonstrate toxicity against WI- 26VA4 cell line (LC_50_ > 100 µM) (Table 1), comparable to that of chloroquine and artemether, standard antimalarials. Compounds AH3, AH6 and AH7 were not active against the W2 strain, they had results greater than 1 μM, falling outside the established standard IC_50_ < 1 μM [45]. Thus, in the table it was indicated as not determined. The SI was obtained through the relationship between the cytotoxic and antiplasmodic activity of each compound, making it possible to identify that the compounds are selective. These findings are in accordance with a set of criteria for the development of antimalarial drugs [45].

Among the in vitro results, the AH1, AH4 and AH5 compounds showed the best IC_50_ values of 0.09 μM and 0.07 μM, respectively, and high SI of > 100. These findings corroborate the criteria for developing new antimalarial compounds announced in the literature [45].

### Molecular docking

The binding energy is directly associated with the conformation adopted by the ligand inside the active site of the protein. Binding energy values lower than that of the crystallographic ligand are attributed to conformations that generate a more stable complex between the ligand of interest and the receptor. The evaluation of these energy values may suggest the most active compounds, as lower binding energy values suggest greater affinity between the compound and the tested target [25].

The energy values generated by the AutoDock Vina software program (Table 2—indexed at the end of the manuscript) demonstrated that the AH1 compound did not show the lowest binding, AH5 compound had the lowest binding energy values (between − 9.7 and − 0.3 kcal/mol) among the tested compounds for 23 targets. On the other hand, the AH4 compound had the lowest binding energy (between − 9.7 and − 8.7 kcal/mol) for 3 targets.

**Table 2:** Binding energy values of compounds *N*-acylhydrazone derivatives

Binding energy (kcal/mol)									
Target	AH1	AH2	AH3	AH4	AH5	AH6	AH7	Crystallographic ligand	Enzymatic class
1LF3	− 6.6	− 6.6	− 6.7	7.0	− 8.0	− 7.7	− 6.2	− 9.6	Hydrolase
1LYX	– 6.2	– 5.6	– 5.9	– 6.3	– 6.0	– 7.6	– 5.7	− 5.6	Isomerase
1NHW	– 7.3	– 7.7	– 7.7	– 7.5	– 9.1	– 8.9	– 7.1	− 8.3	Oxidoreductase
1O5X	− 5.1	− 5.3	− 5.5	− 5.6	− 6.3	− 5.9	− 5.1	− 1.0	Isomerase
1QNG	− 5.9	− 6.0	− 6.0	− 6.2	− 8.1	− 7.6	− 5.8	− 7.7	Isomerase
1RL4	− 6.1	− 6.1	− 6.1	− 6.3	− 6.8	− 6.9	− 5.8	− 8.0	Hydrolase
1TV5	− 9.2	− 8.3	− 8.1	− 8.6	− 7.1	− 7.9	− 7.9	− 9.3	Oxidoreductase
1U4O	− 6.2	− 6.5	− 7.0	− 6.6	− 7.4	− 6.8	− 6.2	− 8.1	Oxidoreductase
1YWG	− 7.0	− 7.1	− 7.3	− 7.3	− 7.6	− 7.1	− 6.7	− 10.7	Oxidoreductase
2AAW	− 6.2	− 6.4	− 6.2	− 6.3	− 7.0	− 7.0	− 5.7	− 9.1	Transferase
2ANL	− 6.9	− 7.2	− 7.3	− 6.9	− 7.8	− 7.4	− 6.6	− 9.3	Hydrolase
2OK8	− 4.7	− 4.7	− 5.3	− 4.9	− 5.6	− 5.8	− 4.6	− 2.0	Oxidoreductase
2PML	− 7.8	− 8.1	− 8.5	− 8.1	− 8.6	− 8.6	− 7.7	− 6.9	Transferase
2Q8Z	− 7.0	− 6.8	− 6.9	− 7.6	− 5.9	− 5.3	− 7.3	− 9.7	Lyase
2VFA	− 5.9	− 5.9	− 6.5	− 5.9	− 6.5	− 6.6	− 5.7	− 1.0	Transferase
2VN1	− 7.8	− 8.0	− 7.9	− 7.7	− 9.7	− 9.2	− 7.5	− 14.6	Isomerase
2YOG	− 8.5	− 7.7	− 7.8	− 8.8	− 8.9	− 8.5	− 7.8	− 8.4	Lyase
3AZB	− 0.2	− 0.3	− 0.3	− 0.3	− 0.3	− 0.2	− 0.3	− 1.0	Hydrolase
3BPF	− 6.3	− 5.9	− 6.2	− 5.8	− 7.0	− 7.4	− 5.8	− 6.3	Signaling Protein
3CLV	− 8.1	− 8.2	− 8.2	− 8.0	− 8.2	− 8.7	− 8.2	− 11.7	Hydrolase
3FNU	− 6.7	− 7.1	− 7.1	− 6.8	− 7.9	− 7.9	− 6.4	− 9.2	Transferase
3K7Y	− 7.5	− 7.7	− 7.4	− 7.6	− 8.5	− 7.8	− 7.4	− 7.7	Lyase
3N3M	− 7.4	− 6.7	− 8.0	− 8.7	− 5.7	− 6.0	− 7.8	− 9.6	Transferase
3PHC	− 9.4	− 9.1	− 8.6	− 9.7	− 8.2	− 7.7	− 8.8	− 8.3	Hydrolase
3QS1	− 7.5	− 7.7	− 7.2	− 7.2	− 9.0	− 8.1	− 6. 7	− 10.4	Hydrolase
3T64	− 7.0	− 7.4	− 7.4	− 7.0	− 8.4	− 7.8	− 6.6	− 8.1	Transferase
4B1B	− 7.7	− 7.4	− 7.4	− 7.9	− 9.7	− 9.1	− 7.7	− 12.3	Oxidoreductase
4C81	− 5.6	− 5.7	− 5.6	− 5.9	− 6.9	− 6.6	− 5.5	− 1.0	Lyase
4J56	− 7.7	− 7.6	− 7.4	− 7.9	− 9.5	− 9.5	− 7.8	− 13.0	Oxidoreductase
4N0Z	− 5.5	− 5.5	− 5.4	− 5.3	− 6.1	− 5.7	− 5.3	− 1.0	Oxidoreductase
4P7S	− 5.7	− 5.6	− 6.0	− 6.0	− 6.9	− 6.0	− 5.4	− 6.0	Cytokine Inhibitor
4QOX	− 8.0	− 8.1	− 7.7	− 7.8	− 9.3	− 8.3	− 7.8	− 8.9	Transferase
PfATP6	− 7.8	− 7.4	− 7.4	− 7.7	− 8.4	− 7.4	− 7.4	− 7.2	Transporter
PfHT	− 7.3	− 7.2	− 7.5	− 7.4	− 8.4	− 8.8	− 7.2	− 5.7	Transporter

Among the targets in which the AH5 compound was the most active, it also obtained lower energy than the crystallographic ligand in only 12 of them. The AH4 compound had lower binding energy than the crystallographic one in only one of the targets in which it was the compound with the highest affinity. Due to the random character of the AutoDock Vina search algorithm, the results were performed in triplicate (Additional File 8: Data S1), suggesting that it is robust for virtual screening experiments.

The mechanism of action of acylhydrazones is still uncertain, and previous studies suggest that these compounds act by inhibiting malaria cysteine proteases, such as falcipain-2 [46]. However, analysis of the docking of our derivatives for falcipain-2 (represented in BraMMT by the target 3BPF) [47] showed that the most active compound for the target is AH6 (binding energy value of − 7.4 kcal/mol), which showed a high IC_50_ in vitro.

The binding energy of the AH5 compound was less than that of the other compounds and that of the crystallographic ligand in the following proteins: 1NHW [48], 1O5X [49], 1QNG [50], 2PML [51], 3K7Y [52], 3T64 [53], 4B1B [54], 4C81 [55], 4N0Z [56], 4P7S [57], 4QOX [58], PFATP6 [59]. A possible explanation for this result is the presence of the benzene ring as the R substituent of the AH5 compound. The increase in non-polar groups in the molecule can cause a reduction in binding energy by up to − 1.0 kcal/mol per van der Waals bond, thus directly influencing the greater affinity of the compound; however, selectivity may vary depending on the presence of hydrophobic cavities in the region of the target’s active site [61, 62].

**Fig. 1:**
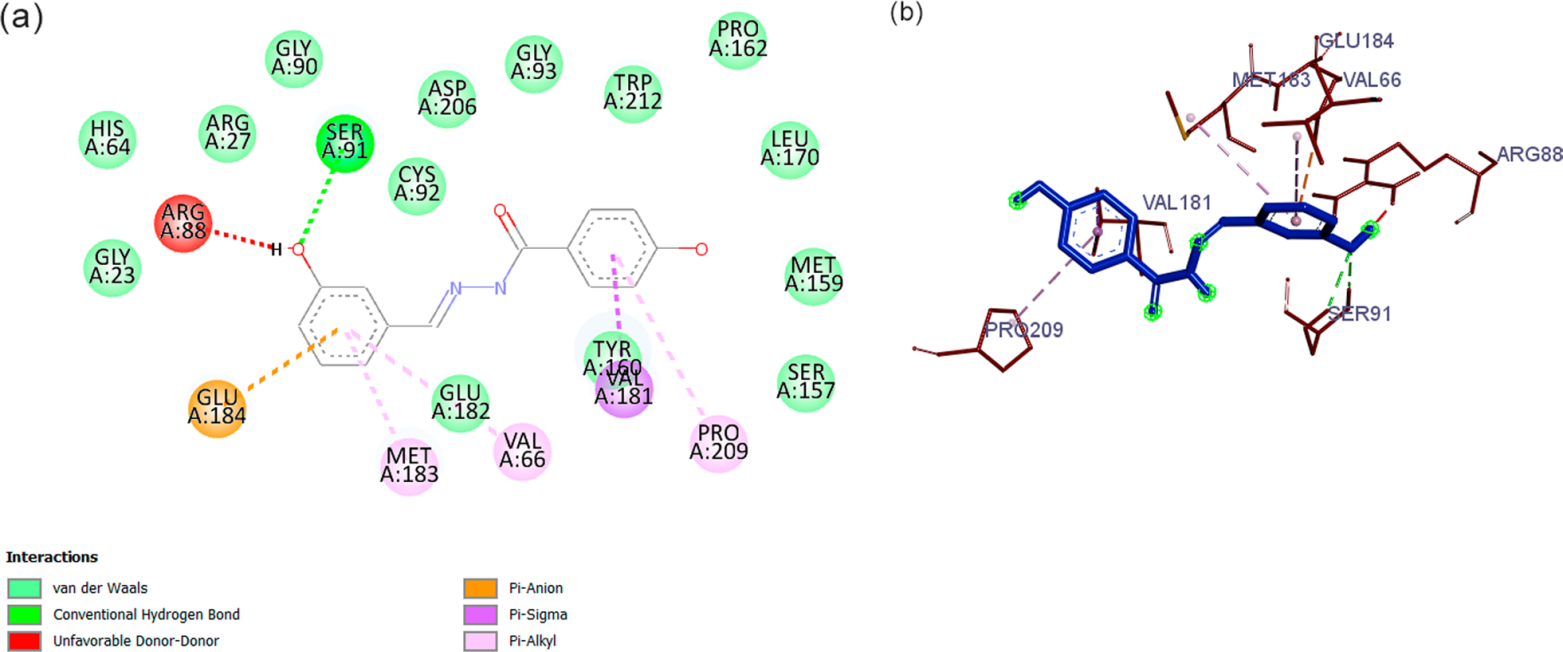
Intermolecular interactions profile of the AH4 compound. **a** Pharmacophoric map, **b** 3D representation of the 3PHC active site: AH4 compound (blue) and 3PHC amino acids (red)

The AH4 compound showed more affinity for only one target: 3PHC, having the lowest binding energy for that target with a binding energy of − 9.7 kcal/mol, compared to − 8.3 kcal/mol for the 3PHC crystallographic ligand, and it also showed an energy difference of − 1.7 kcal/mol for that target. Thus, an analysis of the interaction profile of the AH4 compound with the 3PHC target was made. The intermolecular interactions (Fig. 1) of the complex between the AH4 compound and the 3PHC target was generated by the Discovery Studio Visualizer software program*.*

The 3PHC target corresponds to *P. falciparum* purine phosphorylase (PfPNP) [63, 64]. This enzyme acts in the DNA synthesis pathway of the parasite through phosphorylating purines captured from the human erythrocyte. Studies using both inhibitory compounds, as well as genetic disruption, have shown that blocking PfPNP activity causes a defect in *P. falciparum* growth, mainly in the trophozoite phase where the greatest activity of this enzyme as the protozoan replicates its genome and produces merozoites [63].

An analysis of the pharmacophoric map of the AH4 compound with PfPNP showed a hydrogen bond of the phenol group with a Ser96, in addition to interactions of the Pi-Anion and Pi-Alkyl type between Glu184, Val66 and Met183 and the phenolic ring. Other van der Waals interactions also occurred with a Pro209 and Val181. The phenolic group observed an unfavorable interaction with Arg88, which could negatively influence affinity. Therefore, the set of other interactions attained proved to be compensatory. In view of this, more robust in silico studies, such as the use of molecular dynamics, are needed to assess the stability of the complex formed [64].

### SwissADME

The oral administration route is the most recommended in developing drugs for tropical diseases, because it is considered easy for patients to adhere to treatment; therefore, it is necessary for the drugs to have good oral bioavailability they can be linked to good solubility and essential permeability to predict the ability of compounds to reach their target at established concentrations [65, 66].

Ndombera concluded that an in silico analysis on the absorption, distribution, metabolism, excretion and toxicity of the compound (ADMET) can reduce failures related to pharmacokinetics in the clinical phase in drug development. Furthermore, it is a low cost in silico approach [67]. In view of the in vitro test, the AH4 and AH5 compounds which presented the best IC_50_ values of 0.09 μM and 0.07 μM, respectively, were evaluated under the ADMET parameters. Next, the AH3 compound, which presented the worst result in the in vitro test, and the standard chloroquine and artemether antimalarials were tested in the SwissADME software program (http://www.swissadme.ch/) to compare and validate the SwissADME study, shown in Table 3.

**Table 3:** SwissADME results of compounds derived from *N*-acylhydrazones

Compounds	MW < 500	HA < 10	HD < 5	Log P < 5	TPSA < 140 Å	Caco-2 (cm/s)	LR < 9	Log S > − 5
AH3	285.25	5	2	1.63	107.51	0.55	5	− 3.61
AH4	256.26	4	3	1.18	81.92	0.55	4	− 2.80
AH5	359.38	4	3	2.5	90.79	0.55	7	− 4.18
Chloroquine	305.85	2	1	3.68	28.16	0.56	8	− 4.21
Artemether	298.37	5	0	3.19	46.15	0.55	1	− 3.85

*MW *molecular weight, *HA* hydrogen bond acceptors, *HD* hydrogen bond donors, *log P* partition coefficient, *TPSA* polar topological surface area, *Caco-2* Human Colon Carcinoma Cell Line, *LR* rotating bond, *Log S* solubility

In this study, the criteria of the rule of five by Lipinski were evaluated, which aims to assess the similarity with a drug, the possibility of becoming an active oral medication and the physical–chemical properties [68]. To do so, the compounds must be within the parameters: molecular weight (MW) less than 500 mg/dL, no more than 5 hydrogen bond donors (HD), no more than 10 hydrogen bond acceptors (HA), octanol-partition coefficient water (log P) not greater than 5 [21].

Other criteria were evaluated such as the polar topological surface area (TPSA) less than 140 Å, solubility in water (log S) having a value < − 5, the number of rotating connections (LR) less than < 9 [21]. The bioavailability score analyzed in the human colon carcinoma cell line in the value of 0.55 cm [21]. With these criteria within the established parameters, a compound with a good permeability to membranes is provided, indicating that the compound might have better absorption [69, 70].

Based on Table 3, compounds derived from *N*-acylhydrazones obtained results according to the Lipinski criteria, molecular weight 256.26–359.38 g/mol, number of hydrogen bond acceptors 2–5, number of hydrogen donors 0—3, clogP of 1.18–3.68. Lipophilicity is an important feature in the drug absorption and elimination process along with and a solubility in water which ranged from − 2.80 to − 4.18 mol/L, representing the compounds as soluble in water. The polar topological surface area (TPSA) values of the compounds did not exceed the parameters ranging from 28.16–107.51 Å, indicating that they will have significant permeability in the cell plasma membrane. Rotating connections (LR) ranged from 1–8, not causing a negative effect on the permeation rate. The bioavailability score ranged between 0.55–0.56 cm, which means good pharmacokinetic property [71, 72].

Nugraha has demonstrated the importance of in silico evaluation using the SwissADME software program (http://www.swissadme.ch/) in order to analyze the efficacy of test compounds in predicting the pharmacokinetic properties for oral administration [73].

The results for the AH3, AH4, AH5 compounds and test compounds revealed that the compounds did not violate any of the standards established by ADMET, so they can be administered orally [21, 68–70]. Furthermore, correlating the results obtained in vitro with in silico, the AH4 compound showed agreement with the standards to be formulated as a good promising compound [68].

## Conclusion

Considering the in vitro methods for evaluating the antiplasmodic and cytotoxic activity and in silico molecular modeling. The AH1, AH2, AH4 and AH5 compounds showed interesting properties for the continuation of the study, although experimental tests still need to be carried out to confirm the proposed mechanism of action and determine the structure–activity relationship of the compounds. Compounds AH4 and AH5 are being evaluated in an in vivo test, data that will be complementary to the in silico data presented.

## Experimental

### Chemistry

#### General methods

All reagents and solvents were purchased from Sigma (São Paulo, Brazil) and were used as such, except for the E1-E6 esters which were part of a household collection (Additional File 8: Data S1). The reaction courses were monitored by thin-layerchromatography (TLC) on silica gelG plates (Macherey–Nagel, DC-Fertigfolien ALUGRAM^®^ XtraSil G/UV254) and different mixtures of hexanes/ethyl acetate as the eluents. Column grade silica gel (Sorbiline; 0.040–0.063 mm mesh size) was employed for column chromatography. Melting points of the compounds were obtained on a Bücher 535 melting-point apparatus and are uncorrected. Infrared (IR) spectra were recorded on a Shimadzu FTIR-Affinity-1. Nuclear Magnetic Resonance (NMR) spectra were recorded on a Brucker DPX 200 spectrometer (Rheinstetten, Germany) (200 MHz for 1H NMR and 50 MHz for 13C NMR spectra) in deuterated dimethylsulfoxide (DMSO-d6). Chemical shifts (δ) were reported in parts per million (ppm) with reference to tetramethylsilane (TMS) as internal standard and coupling constants (J) were reported in Hertz (Hz). The LC–MS analysis were performed on an Acquity UPLC-H class system from Waters comprised of quaternary solvent manager attached to a triple-quadrupole (Acquity TQD) mass spectrometer. The output signals were monitored and processed using the Empower 3 software program. The samples were prepared in acetonitrile. Water spiked with 1% with trifluoroacetic acid and acetonitrile at the ratio 1:99 (v/v) was used as mobile phase. High-resolution mass spectrometry (HRMS) was recorded using an ESI micrOTOF-QII Bruker mass spectrometer.

### General method for the synthesis of hydrazides H1-H6

The respective ester (1 eq) and hydrazine hydrate 80% (5 eq) were stirred in etanol (25 mL) under reflux for 8–48 h. Then, the reaction mixtures were cooled to 20–25 °C and then kept at 2–8 °C for 24 h. The solid hydrazides were collected by vacumn filtration, purified by recrystallization in hot ethanol and used as such for the next reactions. The identities of the hydrazides were confirmed by comparison of their IR and NMR spectra to the literature data and were in full agreement [34–38, 74].

### General method for the synthesis of acylhydrazones AH1- AH7

The respective hydrazide (1 eq), 3-hydroxybenzaldehyde (1 eq) and concentrated HCl (2 drops) were stirred in etanol (20 mL) at 20–25 °C for 8 h. The acylhydrazones were obtained as crystaline solids after vacuum filtration and recrystallization with hot ethanol.

*N'-[(1E)-(3-hydroxyphenyl)methylene]benzohydrazide* (**AH1**, C_14_H_12_N_2_O_2_):
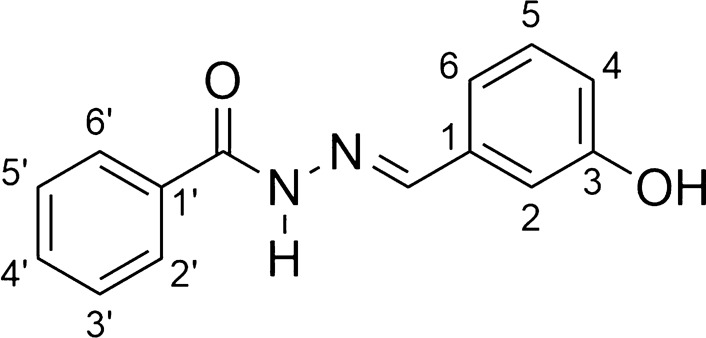


Yellowish solid; yield 91%; melting point: 195–196 °C. 1H NMR (200 MHz, DMSO-d6) d 11.78 (s, 1H, NH), 9.61 (s, 1H, OH), 8.37 (s, 1H, N = CH), 7.91 (d, 2H, H-2’ and H-6’, J = 5.6 Hz), 7.61–7.50 (m, 3H, H-3’, H-4’, H-5’), 7.27–6.82 (m, 4H, H-2, H-4, H-5 and H-6); 13C NMR (50 MHz, DMSO-d6) d 163.5 (C = O), 158.1 (C-3), 148.3 (N = CH), 136.0 (C-1), 133.9 (C-1’), 132.1 (C-4’), 130.3 (C-5), 128.9 (C-3’and C-5’), 128.0 (C-2’ and C-6’),119. 2 (C-6), 117.9 (C-4), 113.0 (C-2). *4-chloro-N'-[(1E)-(3-hydroxyphenyl)methylene]benzohydrazide* (**AH2**, C_14_H_11_Cl N_2_O_2_):
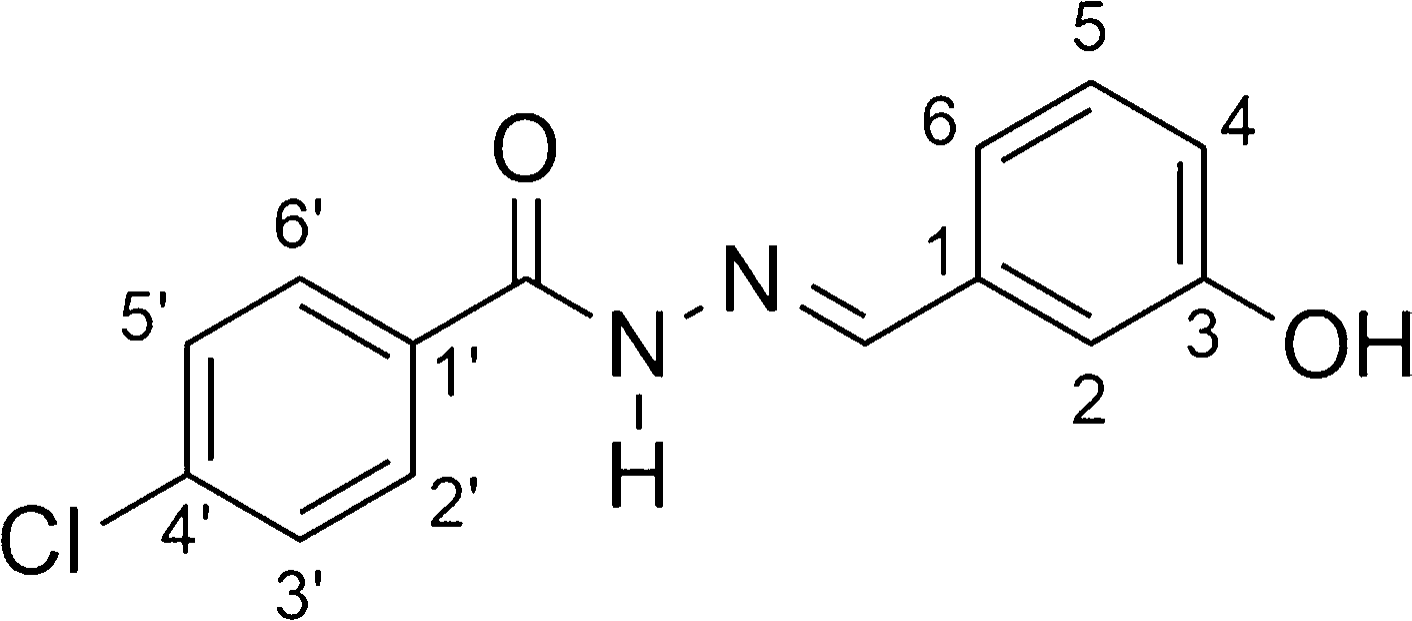


White solid; yield 85%; melting point: 221–223 °C.IR (ATR) ῡmax/cm^−1^: 3277 (v N–H), 3099 (v C-H ar), 1647 (v C = O), 1560 (v C = N), 1275 (v C-O); 1H NMR (200 MHz, DMSO-d6) d 11.82 (s, 1H, NH), 9.59 (s, 1H, OH), 8.34 (s, 1H, N = CH), 7.91 (d, 2H, H2’ and H-6’, J = 6.12 Hz), 7.58 (d, 2H, H-3’ and H-5’, J = 6.12 Hz), 7.23–6.80 (m, 4H, H2, H-4, H-5 and H-6); 13C NMR (50 MHz, DMSO-d6) d 161.4 (C = O), 157.1 (C-3), 147.6 (N = CH), 136.0 (C-1), 134.9 (C-4’), 131.6 (C-1’), 129.3 (C-5), 129.0 (C-2’ and C6’), 128.0 (C-3’ and C-5’), 118.3 (C-6), 117.0 (C-4), 112.1 (C-2). MS (ESI) m/z calcd for C14H11ClN2O2 (M + H) + : 275.05, found 274.96.

*N'-[(1E)-(3-hydroxyphenyl)methylene]-4-nitrobenzohydrazide* (**AH3**, C_14_H_11_N_3_O_4_):
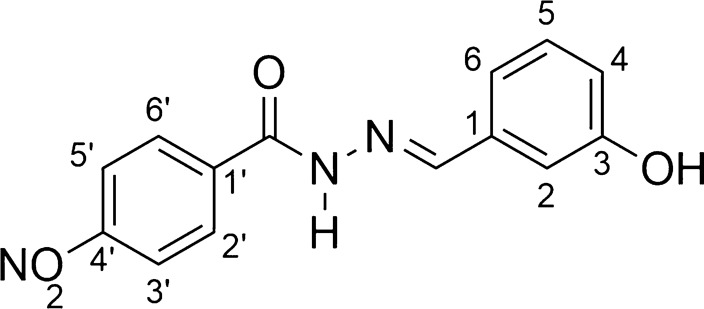


Light gray solid; yield 86%; melting point: 255–256 °C.IR (ATR) ῡmax/cm^−1^: 3280 (v NH), 3051 (v C-H ar), 1661 (v C = O), 1605 (v C = C ar), 1560 (v C = N), 1518 (v NO2), 1348,2 (v NO2), 1282 (v C-O); 1H NMR (200 MHz, DMSO-d6) d 12.04 (s, 1H, NH), 9.61 (s, 1H, OH), 8.36 (s, 1H, N = CH), 8.34 (d, 2H, H-3’ and H-5’, J = 6.3 Hz), 8.13 (d, 2H, H-2’ and H-6’, J = 6.3 Hz), 7.26 (m, 4H, H-2, H-4, H-5 and H-6); 13C NMR (50 MHz, DMSO-d6) d 161.5 (C = O), 156.7 (C-3), 150.5 (C-4’), 139.5 (C-1’), 135.7 (C-1), 130.9 (C-2’ and C-6’), 129.6 (C-5), 124.6 (C-3’ and C-5’), 120.1 (C-6), 118.2 (C-4), 113.6 (C-2).MS (ESI) m/z calcd for C14H11N3O4 (M + H) + : 286.07, found 286.00.

*4-hydroxy-N'-[(1E)-(3-hydroxyphenyl)methylene]benzohydrazide* (**AH4**, C_14_H_12_N_3_O_3_):
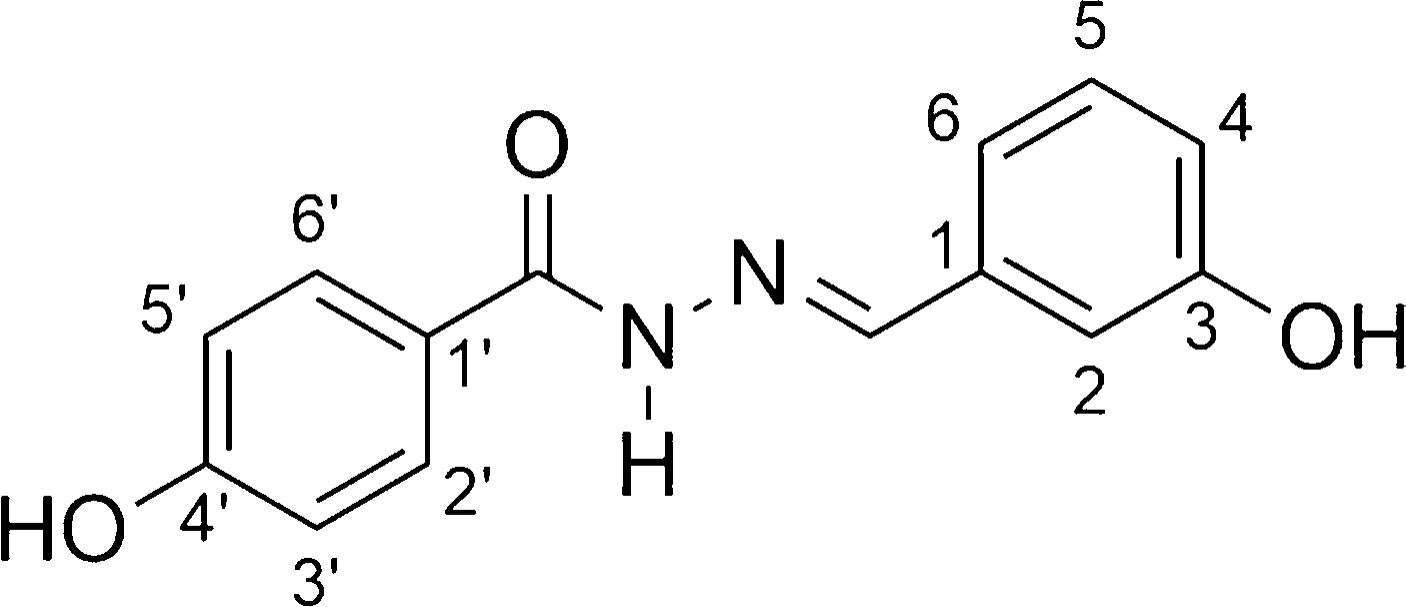


Light brown solid; yield 72%; melting point: 255–258 °C.IR (ATR) ῡmax/cm^−1^: 3200 (v N–H), 3051 (v C-H ar), 1619 (v C = O), 1585 (v C = N), 1236 (v C-O); 1H NMR (200 MHz, DMSO-d6) d 11.61 (NH), 10.22 (OH), 9.69 (OH), 8.31 (N = CH), 7,79 (d, 2H, H2’ and C-6’, J = 8.5 Hz); 13C NMR (50 MHz, DMSO-d6) d 164.5 (C = O), 162.3 (C-4’), 159.8 (C-3’), 147.9 (N = C), 137.4 (C-1), 132.1 (C-5), 131.4 (C-2’ and C-6’), 125.5 (C1’), 121.8 (C-6), 119.6 (C-3’ and C-5’), 116.7 (C- 4), 114.3 (C-2).MS (ESI) m/z calcd for C14H12N2O3 (M + H) + : 257.08, found 256.99.

*N-(4-{[(2E)-2-(3-hydroxybenzylidene)hydrazino]carbonyl}phenyl) benzamide* (**AH5**, C_21_H_17_N_3_O_3_)*:*
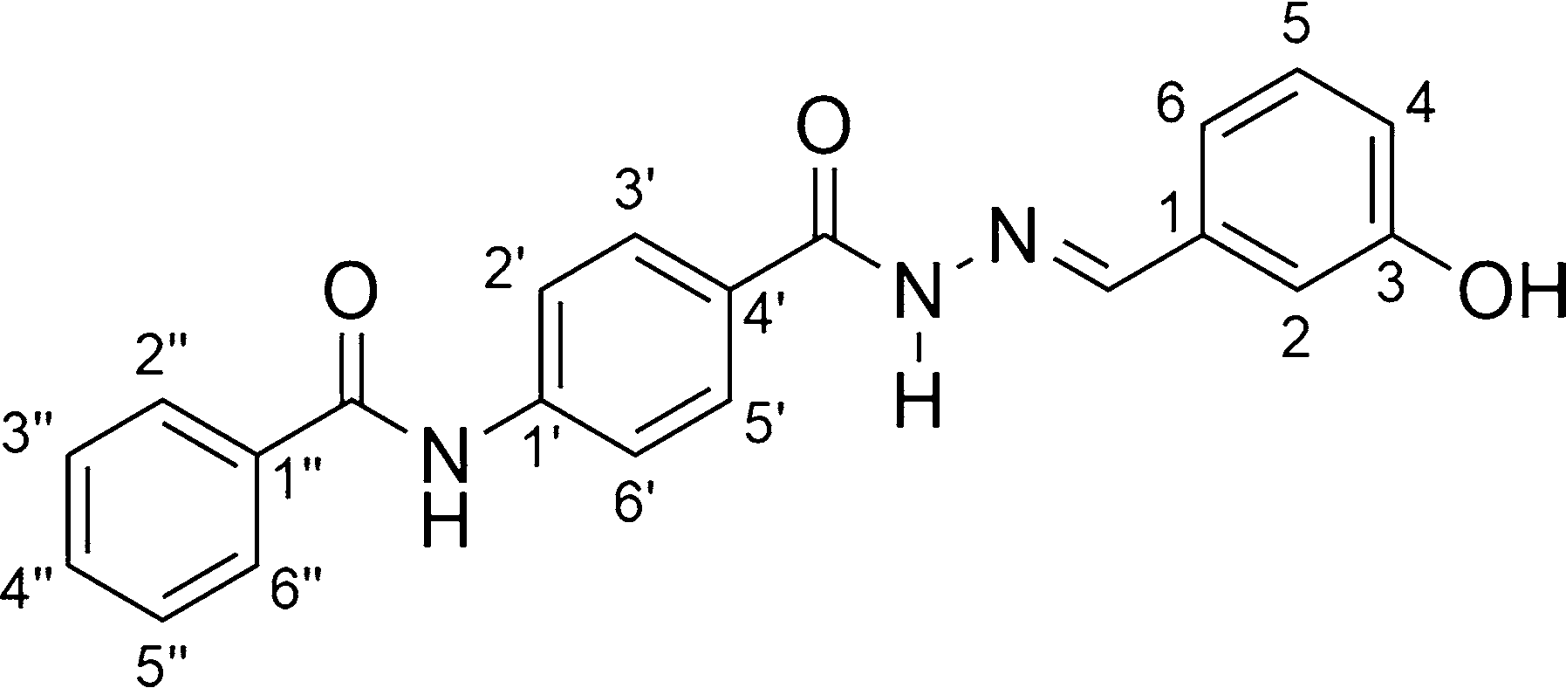


Light gray solid; yield 91%; melting point: 284–286 °C.IR (ATR) ῡmax/cm^−1^: 3423 (v O–H), 3319 (v N–H), 3242 (v N–H), 3079 (v C-H ar), 1651 (v C = O amide), 1633 (v C = O hydrazone), 1511 (v C = N), 1296 (v C-O); 1H NMR (200 MHz, DMSO-d6) d 11.77 (s, 1H, CONHN = C), 10.54 (s, 1H, OH), 9.66 (s, 1H, CONHAr), 8.38 (s, 1H, N = CH), 8.00–6.82 (m, 13H, Harom); 13C NMR (50 MHz, DMSO-d6) d 166.2 (C = O), 162.9 (C = O), 158.0 (C-3), 147.8 (N = C), 142.7 (C-1’), 136.0 (C-1), 135.0 (C-1’’), 132.2 (C-4’’), 130.2 (C-3’ and C-5’), 128.8 (C-3’’ and C-5’’), 128.4 (C-4’), 128.1 (C-2’’ and C-6’’), 119.8 (C-2’ and C-6’), 119.1 (C-6), 117.7 (C-4), 112.9 (C-2).MS (ESI) m/z calcd for C21H17N3O3 (M + H) + : 360.13, found 360.06.

*4-[(4-{[(2E)-2-(3-hydroxybenzylidene)hydrazino]carbonyl}phenyl)amino]-4- oxobutanoic acid* (**AH6**, C_18_H_17_N_3_O_5_):
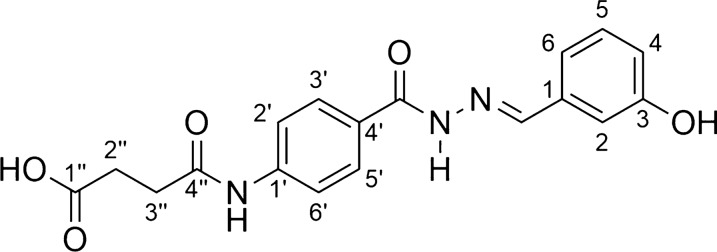


Light gray solid; yield 65%; melting point: 232–234 °C. IR (ATR) ῡmax/cm^−1^: 3400–2400 (v O–H carboxylic acid), 3322 (v N–H), 3242 (v N–H), 3051 (v C-H ar), 1706 (v C = O carboxylic acid), 1665 (v C = O amide), 1644 (v C = O hydrazone), 1522 (v C = N), 1181 (v C-O); 1H NMR (200 MHz, DMSO-d6) d 12.10 (s, 1H, COOH), 11.66 (CONHAr); 10.22 (CONHN = C), 9.59 (s, 1H, ArOH), 8.33 (N = CH), 7.85 (d, 2H, H-2’ and H-6’, J = 8.5 Hz), 7.69 (d, 2H, H-3’ and H-5’, J = 8.5 Hz), 7.25–6.78 (m, 4H, H-2, H-4, H-5 and H-6); 2.58–2.44 (m, 4H, H-2’’, H-3’’); 13C NMR (50 MHz, DMSO-d6)d 173.3 (C-4’’), 170.1 (C-1’’), 161.9 (CONHN), 157.1 (C-3), 146.9 (N = C), 141.8 (C-1’), 135.2 (C-1), 129.3 (C-3’ and C-5’), 128.0 (C-5), 126.9 (C-4’), 118.2 (C-6), 117.6 (C-2’ and C-6’), 116.8 (C-4), 112.0 (C-2), 30.64 (C-2’’), 28.17 (C-3’’).MS (ESI) m/z calcd for C18H17N3O5 (M + H) + : 356.12, found 356.01.

*N'-[(1E)-(3-hydroxyphenyl)methylene]isonicotinohydrazide* (**AH7**, C_13_H_11_N_3_O_2_):
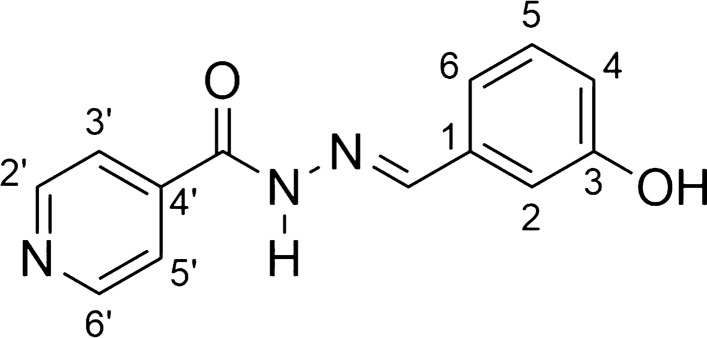


Yellowish solid; yield 93%; melting point: 268–269 °C. IR (ATR) ῡmax/cm^−1^: 3474 (v OH), 3397 (v N–H), 3099 (v C-H ar), 1668 (v C = O), 1561 (v C = N), 1287 (v C-O); 1H NMR (200 MHz, DMSO-d6) d 12.69 (s, 1H,NH), 9.04 (s, 1H, OH), 9.03 (d, 2H, H-2’, H-6’, J = 9.63 Hz), 8.54 (s, 1H, N = CH), 8.36 (d, 2H, H-3’, H-5’, J = 9.63 Hzs), 7.30–6.78 (m, 5H, H-2, H-4, H-5, H-6, OH); 13C NMR (50 MHz, DMSO-d6)d 159.9 (C = O), 154.7 (C-3), 150.4 (C-2’ and C-6’), 146. 2 (N = C), 145.3 (C-4’), 136.1 (C-1), 129.3 (C-5), 123.8 (C-3’ and C-5’), 119.2 (C-6), 118.1 (C-4), 113.0 (C-2).MS (ESI) m/z calcd for C13H11N3O2 (M + H) + : 242.09, found 241.98.

Characterization of all compounds was presented in Additional File 8: Data.

### In vitro* activity of N-acylhydrazones*

*P. falciparum* W2 strain were maintained in continuous culture using human red blood cells in RPMI 1640 medium, supplemented with human plasma [75]. The parasites were synchronized using sorbitol [76] treatment and the parasitemia was microscopically evaluated with Giemsa-stained blood smears.

The blood suspension was adjusted to 2% parasitemia and hematocrit, thus distributed into 96-wells of a microplate containing the *N*-acylhydrazones (AH1, AH2, AH3, AH4, AH5, AH6, AH7), artemether and chloroquine, standard anti-malarial in triplicate for each dose (Additional Files 1, 2, 3, 4 , 5, 6, 7). After 48 h, blood smears were prepared, coded, stained with giemsa and examined at 1000×magnification [77]. The parasitemia of controls (considered as 100% growth) was compared to test cultures, and then the percentage of infected red blood cells was calculated.

The results were expressed as mean of half-maximal inhibitory dose (IC_50_), with different drug concentrations performed in triplicate and compared with drug-free controls. Curve fitting was performed using OriginPro^®^ 8.0 software (Origin Lab Corporation, Northampton, MA, USA).

### Cytotoxicity test

Non-cancerous human lung fibroblast WI-26VA4 (ATCCCCL-95.1) cell line was used to assess the cell viability after each chemical treatment by employing the MTT colorimetric assay [78, 79]. First, 1 × 10^6^ cells were seeded in 96-well microplates with RPMI 1640, the medium supplemented was composed with fetal bovine serum (FBS) and Penicillin Streptomycin antibiotics. Next, microplates were incubated overnight at 37 °C and 5% CO_2_, followed by treatment with *N*-acylhydrazone (AH1, AH2, AH3, AH4, AH5, AH6, AH7), artemether and chloroquine, solubilized in 0.1% DMSO (v/v). Negative controls were composed of cells without treatment. Five serial dilutions (1:10) were made from a stock solution (10 mg mL − 1) using RPMI supplemented with 1% FBS. Cell viability was evaluated after 48 h of incubation by discarding the medium and adding 100 μL of 5% MTT, followed by 3 h of incubation. Then, the supernatant was discarded, and insoluble formazan product was dissolved in DMSO. The optical density (OD) of each well was measured using a microplate spectrophotometer at 550 nm. The OD of untreated control cells was defined as 100% cell viability and all assays were performed in triplicate. The selectivity index (SI) of samples was calculated by following Eq. 1 [29].

1 $$ {\text{SI }} = \frac{{{\text{LC}}_{{{5}0}} {\text{of WI}} - {\text{26VA4 cell line}} }}{{ {\text{IC}}_{{{5}0}} {\text{of}}P. \, falciparum\left( {\text{strain W2}} \right)}} $$

Equation 1 Selectivity index formula.

### Molecular docking

Initially, the structure of the compounds was designed using the Marvin Sketch software program [80]. In this step, protonated form and tautomeric conformers were carefully checked using of hydrogen potential (pH) 7.4 and pH 4.0 [81]. Afterwards, all ligands were refined by semi-empirical Parametric Method 7 (PM7) [82] implemented on MOPAC2012 using the keyword ef for minimum search [83]. Next, refined ligands in pdbqt format were assigned rotatable bonds, Gasteiger– Marsili net atomic charges [84], and hydrogens from polar atoms were kept by the MGLTools software program [85]. The visual inspection of ligands geometry was performed using the Discovery Studio Visualizer software program [86].

After the ligands were prepared, two series of molecular docking calculations were performed using the AutoDock Vina software program (The Scripps Research Institute, La Jolla, CA) [87], coupled to the Octopus platform [88], one with the adjustment of the protonation state of the compounds to pH 7.4, consistent with the pH of the enzymatic environment of most targets, and the other with adjustment to pH 4.0, for the targets 1LF3 [89], 2ANL [90], 3BPF [91], 3FNU [92], 3QS1 [93], which are located in the digestive vacuole of the parasite.

Next, the binding energy generated during molecular docking for each compound was used to evaluate the results. Then, a comparison was made with the energy of the crystallographic ligands of each of the targets. The acylhydrazone derivatives that showed lower energy values in relation to the crystallographic ligand were classified as more active in in silico methods [25].

### SwissADME

The synthetic derivatives of acylhydrazone were designed using Chem Axons Marvins JS (http://www.chemaxon.com), the structures of the compounds were used in the SwissADME software program (http://www.swissadme.ch/) of the Institute of bioinformatics (http://www.sib.swiss) to analyze the profile of the compounds, as well as their results of physical–chemical and pharmacokinetic parameters, and their similarity with drugs were examined [21, 38].

## Supplementary Information

**Additional file 1: Figure SAH1**. N-acylhydrazone compounds AH1.

**Additional file 2: Figure SAH2**. N-acylhydrazone compounds AH2.

**Additional file 3: Figure SAH3**. N-acylhydrazone compounds AH3.

**Additional file 4: Figure SAH4**. N-acylhydrazone compounds AH4.

**Additional file 5: Figure SAH5**. N-acylhydrazone compounds AH5.

**Additional file 6: Figure SAH6**. N-acylhydrazone compounds AH6.

**Additional file 7: Figure SAH7**. N-acylhydrazone compounds AH7.

**Additional file 8: Data S1:** Figures, IR, NMR and MS spectra for AH1-AH7 acylhydrazones

## Data Availability

All data generated or analyzed during this study are included in this published article [and its supplementary information files].

## Abbreviations

ACTs: Artemisinin-based Combination Therapy
ADMET: Parameters of absorption, distribution, metabolism, excretion and toxicity
Arg: Arginine
BraMMT: Brazilian Malaria Molecular Targets
Caco-2: Human Colon Carcinoma Cell Line
DMSO: Dimethylsulfoxide
DMSO-d6: Deuterated dimethylsulfoxide
ESI: Electrospray ionization
FBS: Fetal bovine serum
Glu: Glutamate
HA: Hydrogen bond acceptors
HD: Hydrogen bond donors
Hz: Hertz
IC_50_: Half-maximal inhibitory dose
IR: Infrared
IVS: Inverse virtual screening
J: Coupling constant
LC_50_: Lethal drug concentration that reduced WI-26VA4 cells viability in 50%
log P: Partition coefficient
Log S: Solubility
LR: Rotating bond
Met: Methionine
MS: Mass spectrometry
MW: Molecular weight
NMR: Nuclear magnetic resonance
OD: Optical density
PDB: Protein data bank
P*f*PNP: *P. falciparum* Purine nucleoside phosphorylase
pH: Hydrogen potential
PM7: Parametric Method 7
ppm: Parts per million
Pro: Proline
RPMI: Growth medium used in culture
Ser: Serine; SD: Standard deviation
SI: Selectivity index
TLC: Thin-layerchromatography
TMS: Tetramethylsilane
TPSA: Polar topological surface area
TQD: Triple-quadrupole;
UPLC-H: Acquity UPLC-H class system from WatersAcquity UPLC-H class system fom Waters
Val: Valine
WI-26VA4: Human pulmonary fibroblast cells
W2: Chloroquine-resistant
WHO: World Health Organization
3D7: *Plasmodium falciparum* Chloroquine-sensitive
δ: Chemical shifts
