# Supplementary figures and images for: Evaluation of antiplasmodial activity in silico and in vitro of N-acylhydrazone derivatives
Source: BMC Chem. 2022 Jul 9;16(1):50. doi: 10.1186/s13065-022-00843-9 (PMC9271247; doi:10.1186/s13065-022-00843-9)

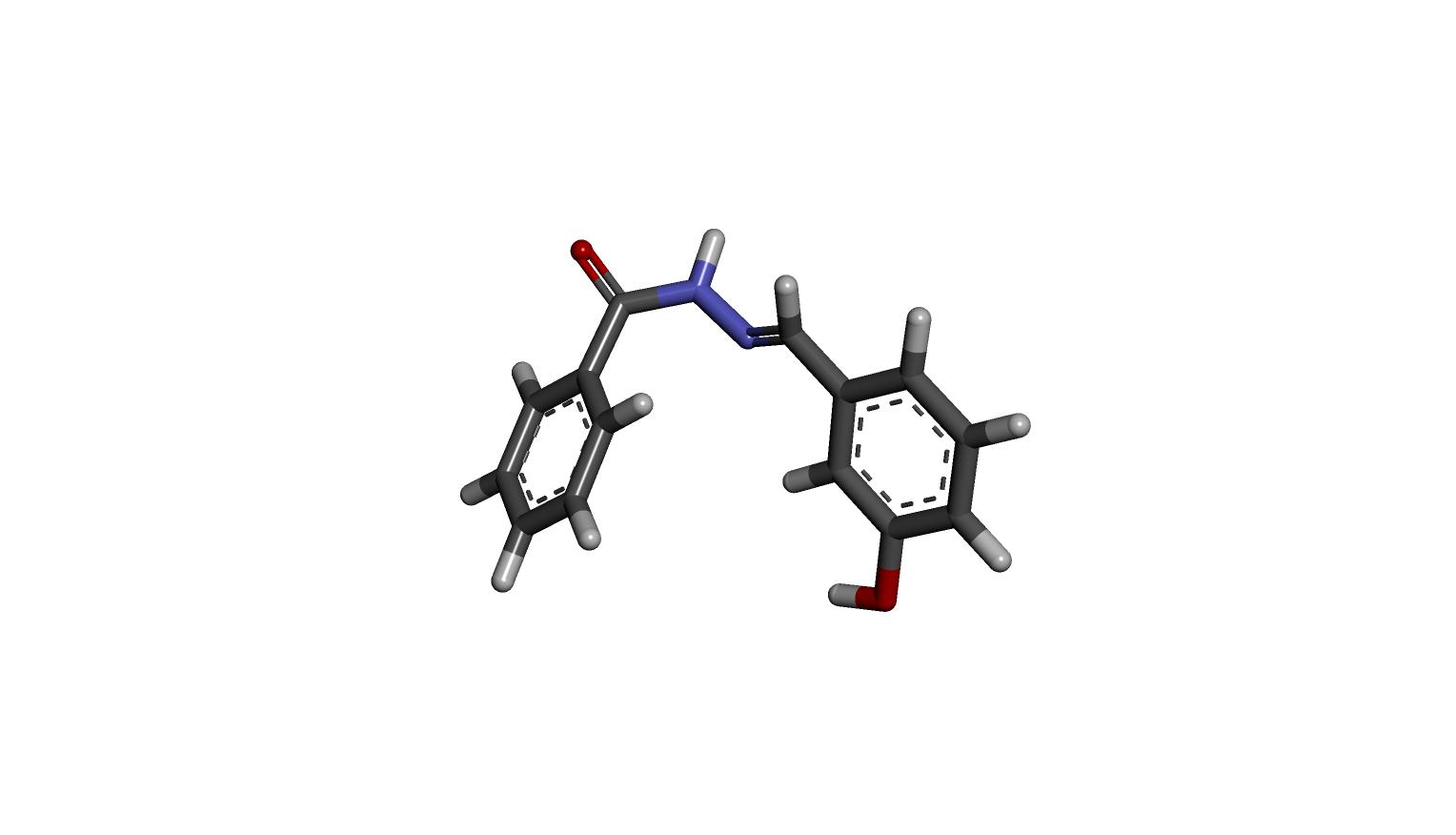

Supplement: Supplementary file 1 — Additional file 1: Figure SAH1. N-acylhydrazone compounds AH1. [file 13065_2022_843_MOESM1_ESM.jpg]

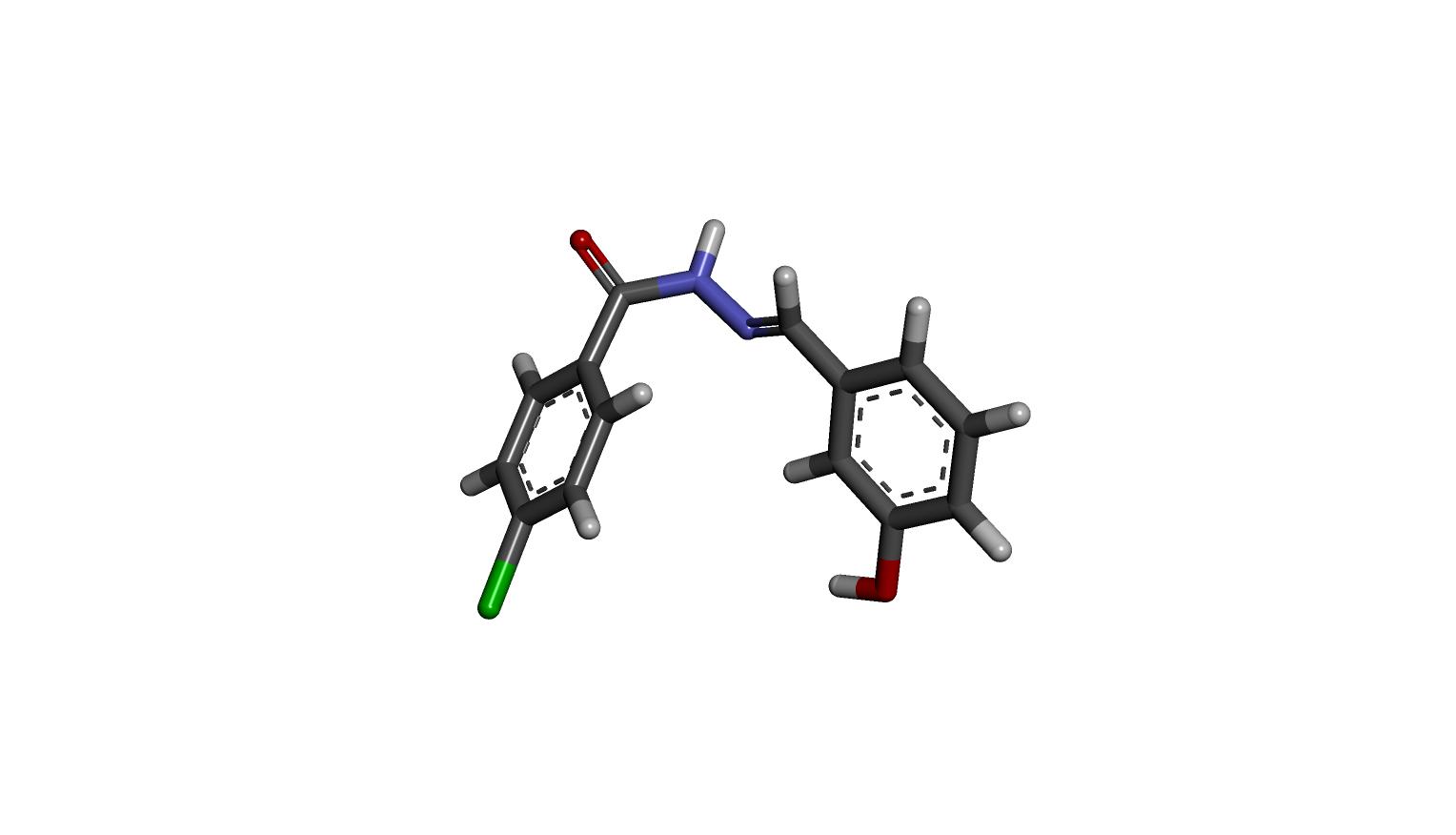

Supplement: Supplementary file 2 — Additional file 2: Figure SAH2. N-acylhydrazone compounds AH2. [file 13065_2022_843_MOESM2_ESM.jpg]

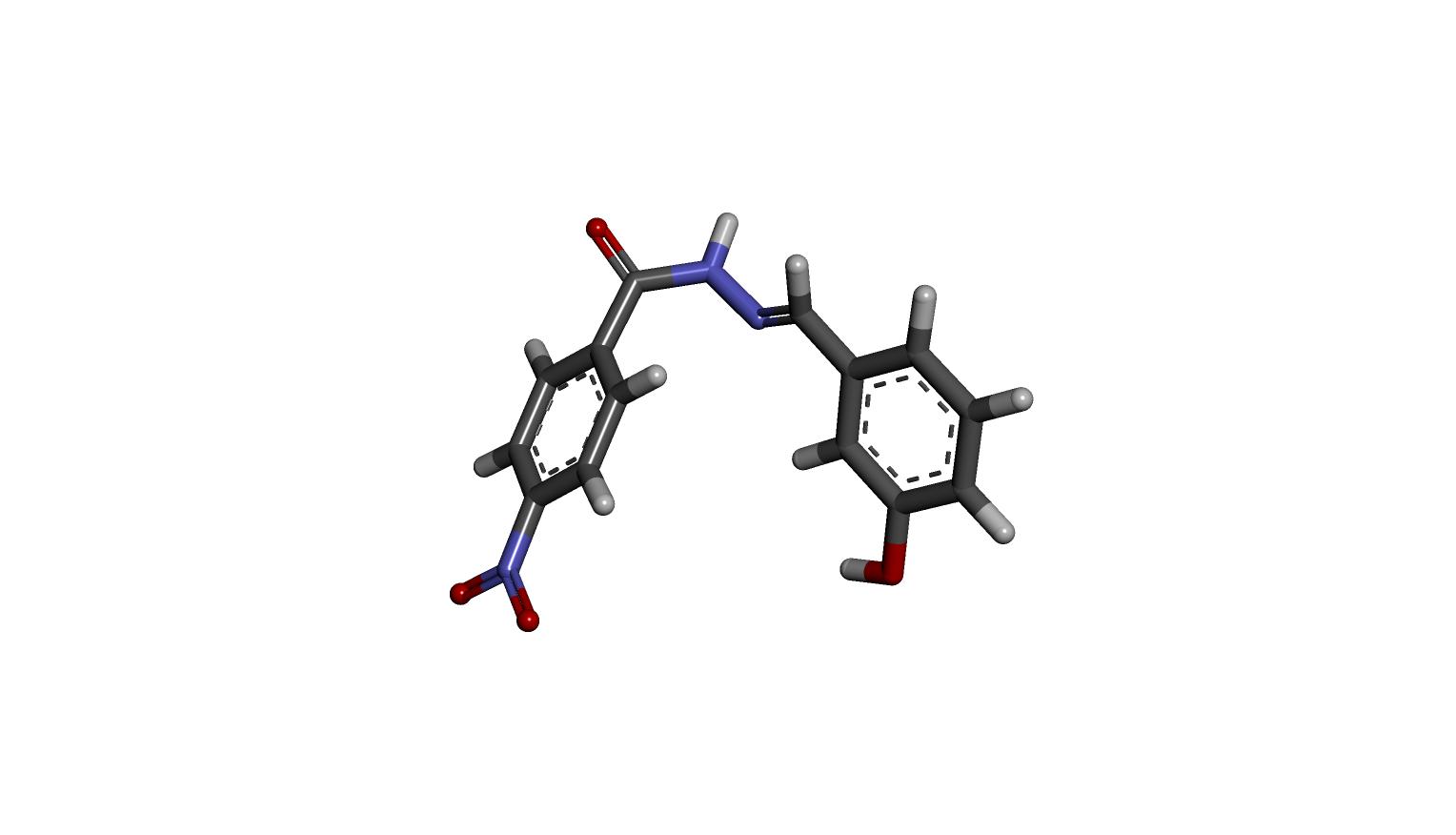

Supplement: Supplementary file 3 — Additional file 3: Figure SAH3. N-acylhydrazone compounds AH3. [file 13065_2022_843_MOESM3_ESM.jpg]

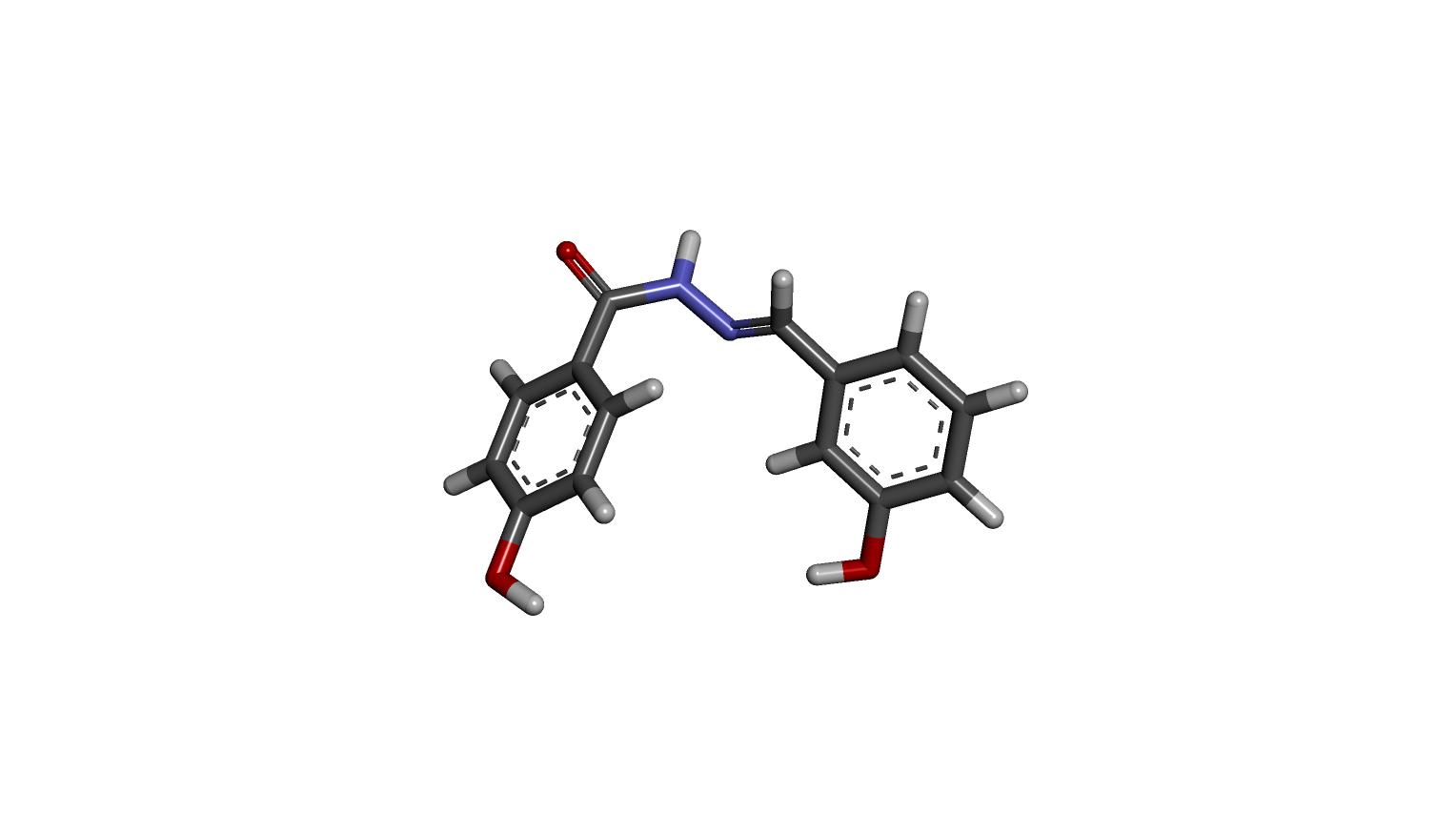

Supplement: Supplementary file 4 — Additional file 4: Figure SAH4. N-acylhydrazone compounds AH4. [file 13065_2022_843_MOESM4_ESM.jpg]

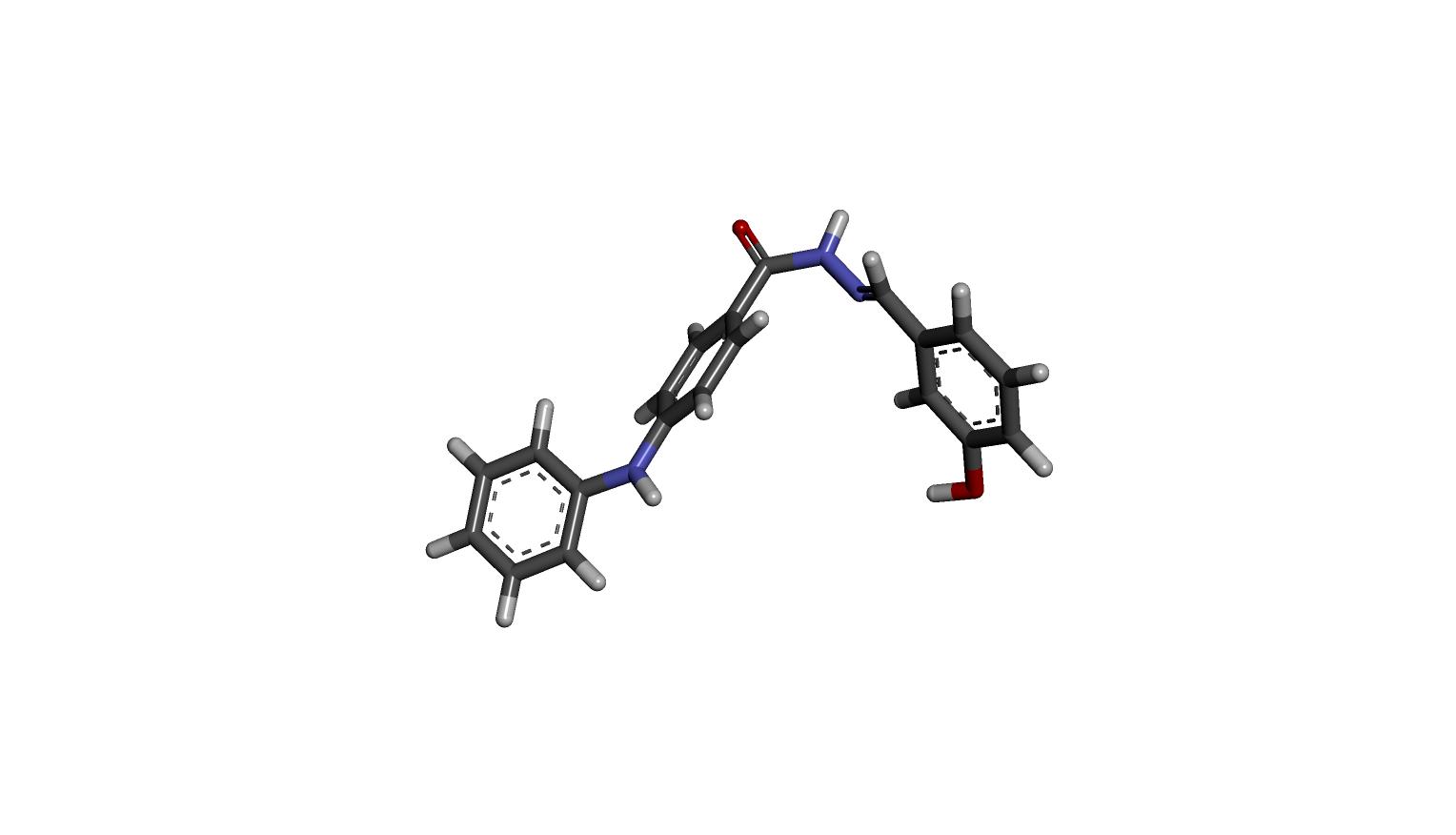

Supplement: Supplementary file 5 — Additional file 5: Figure SAH5. N-acylhydrazone compounds AH5. [file 13065_2022_843_MOESM5_ESM.jpg]

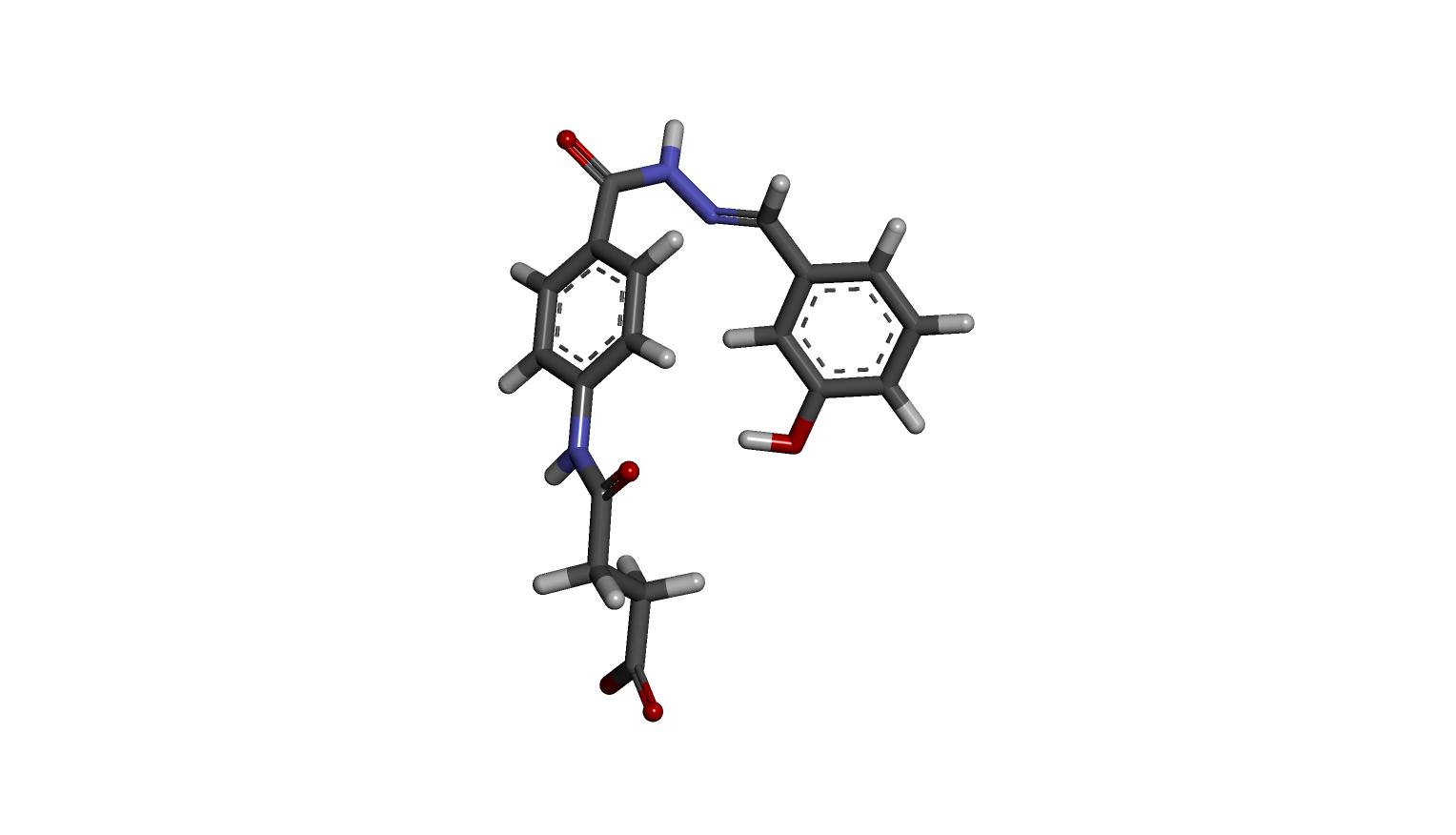

Supplement: Supplementary file 6 — Additional file 6: Figure SAH6. N-acylhydrazone compounds AH6. [file 13065_2022_843_MOESM6_ESM.jpg]

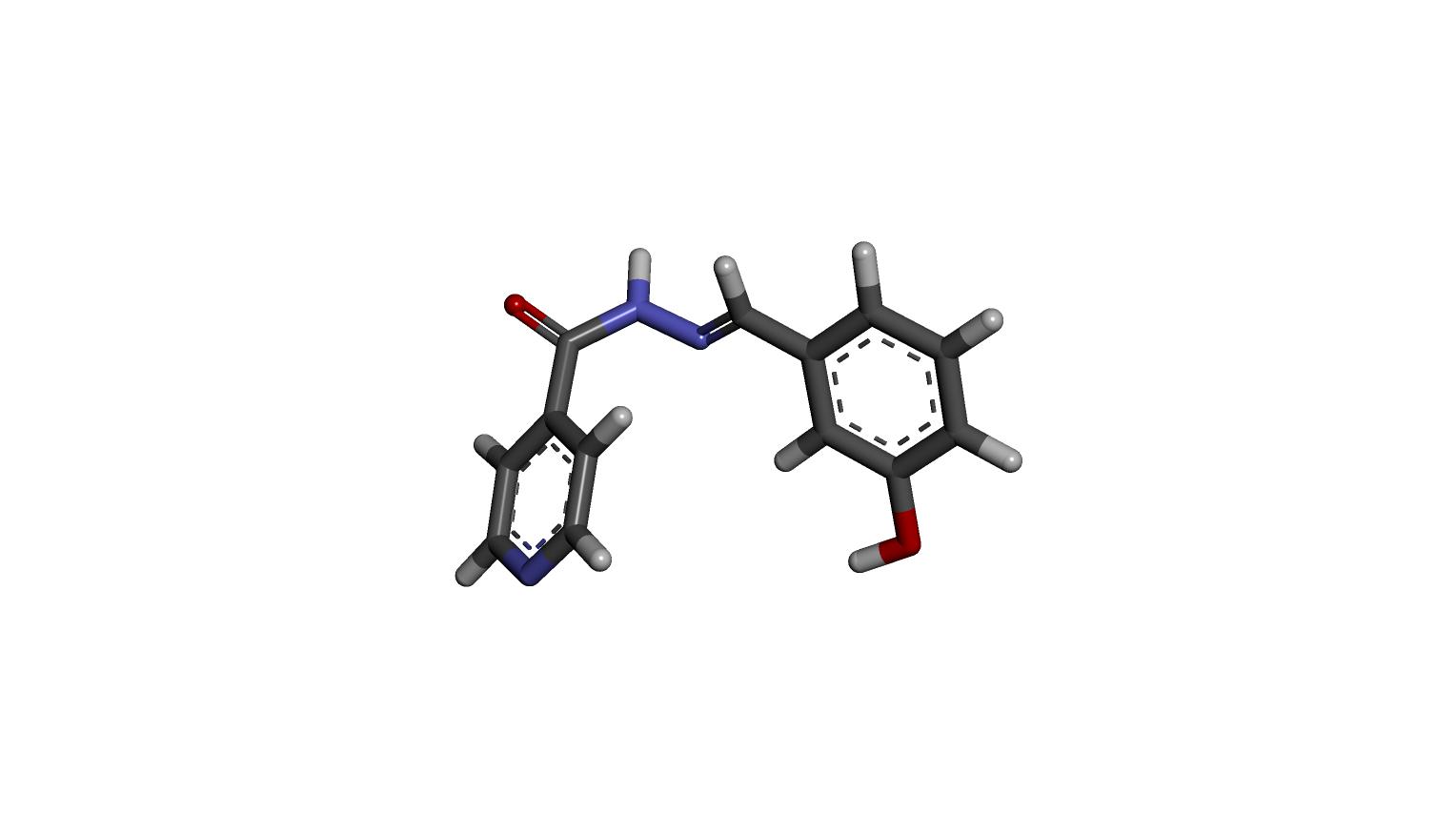

Supplement: Supplementary file 7 — Additional file 7: Figure SAH7. N-acylhydrazone compounds AH7. [file 13065_2022_843_MOESM7_ESM.jpg]
