# Supplementary material for: Evaluation of antiplasmodial activity in silico and in vitro of N-acylhydrazone derivatives
Source: BMC Chem. 2022 Jul 9;16(1):50. doi: 10.1186/s13065-022-00843-9 (PMC9271247; doi:10.1186/s13065-022-00843-9)
Supplement: Supplementary file 8 — Additional file 8: Data S1: Figures, IR, NMR and MS spectra for AH1-AH7 acylhydrazones [file 13065_2022_843_MOESM8_ESM.doc]

**Supplementary Data**

**Figures, IR, NMR and MS spectra for AH1-AH7 acylhydrazones**

**Figure AH1:** N-acylhydrazone compounds AH1.

**Figure AH2:** N-acylhydrazone compounds AH2.

**Figure AH3:** N-acylhydrazone compounds AH3.

**Figure AH4:** N-acylhydrazone compounds AH4.

**Figure AH5:** N-acylhydrazone compounds AH5.

**Figure AH6:** N-acylhydrazone compounds AH6.

**Figure AH7:** N-acylhydrazone compounds AH7.

**Infrared spectra (ATR, ῡ_max_, cm^-1^)**


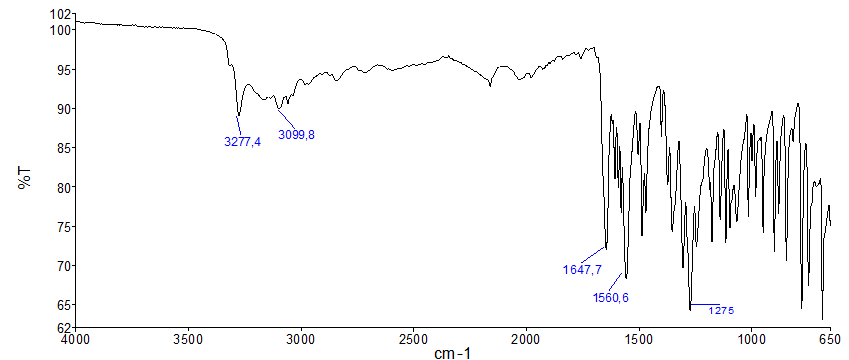


IR spectrum for AH2


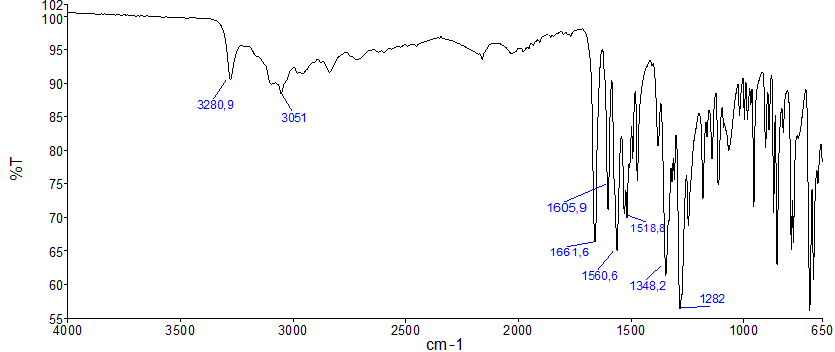


IR spectrum for AH3


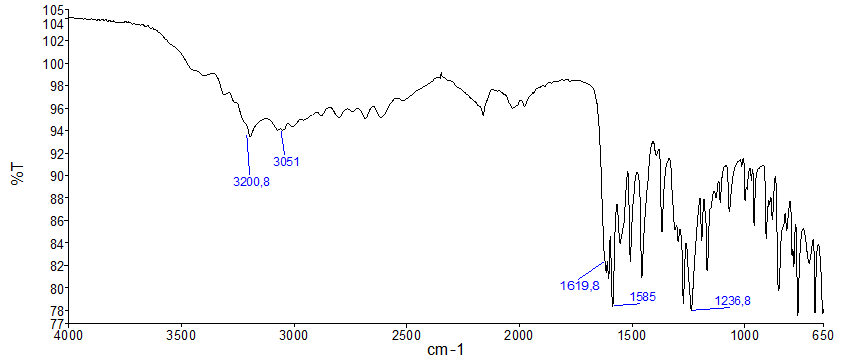


IR spectrum for AH4


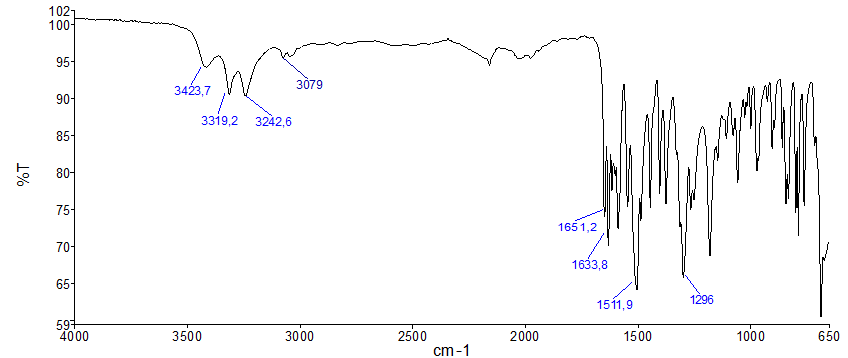


IR spectrum for AH5


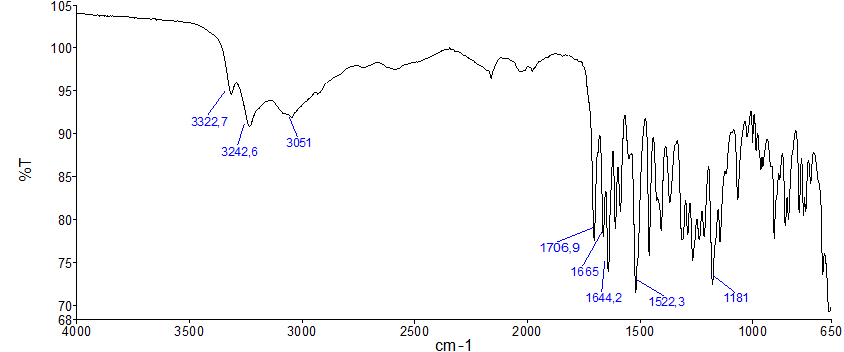


IR spectrum for AH6


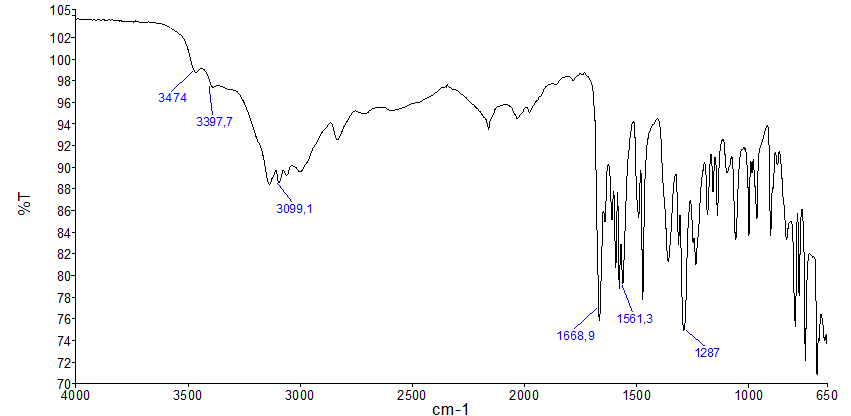


IR spectrum for AH7

**^1^H (300 MHz, DMSO-*d*_6_) and ^13^C (75 MHz, DMSO-*d*_6_) NMR spectra**


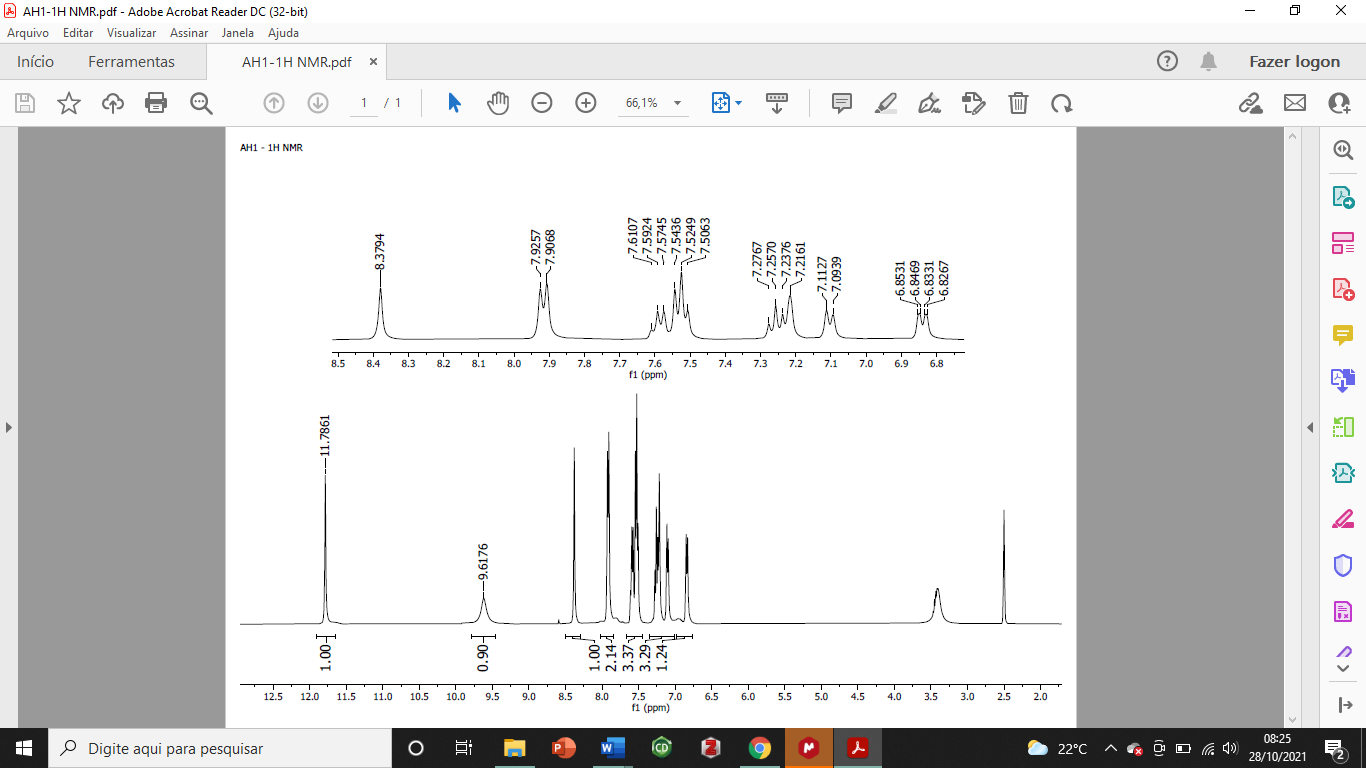


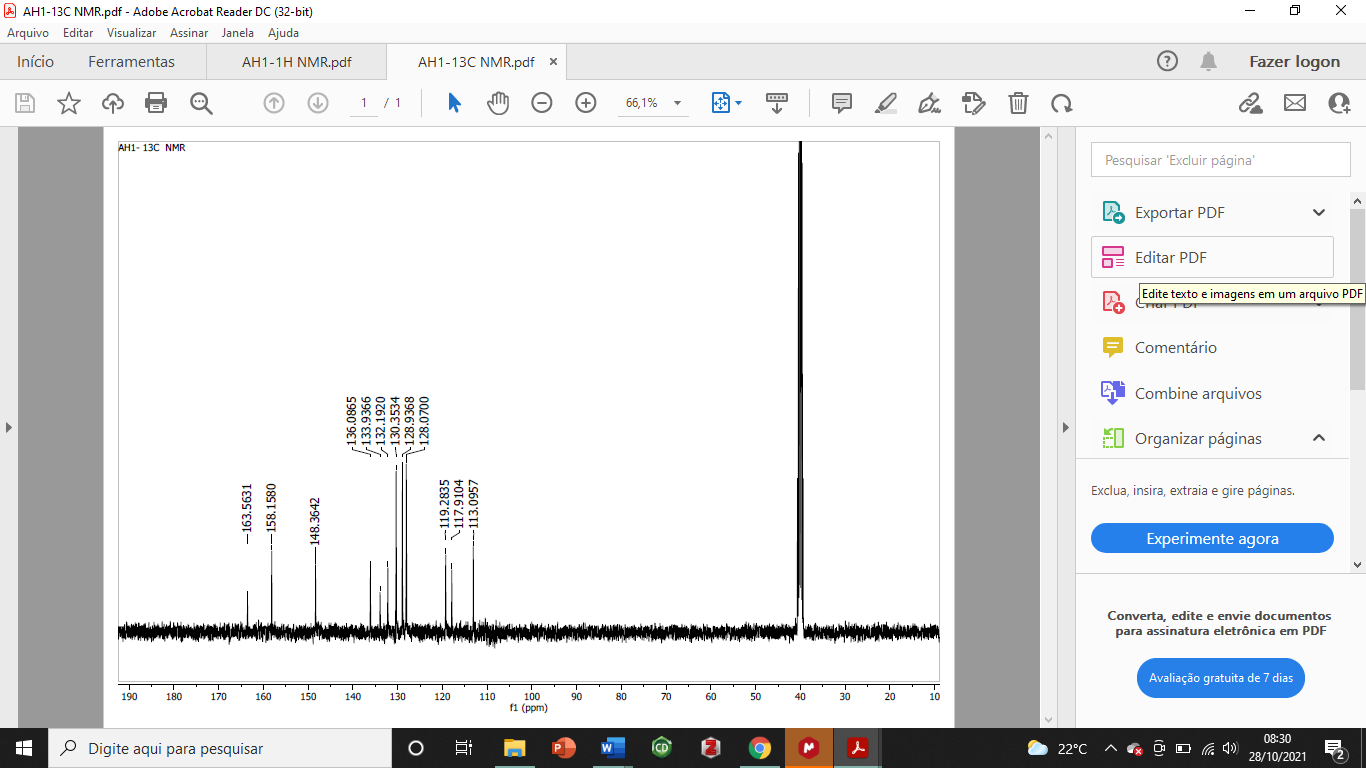


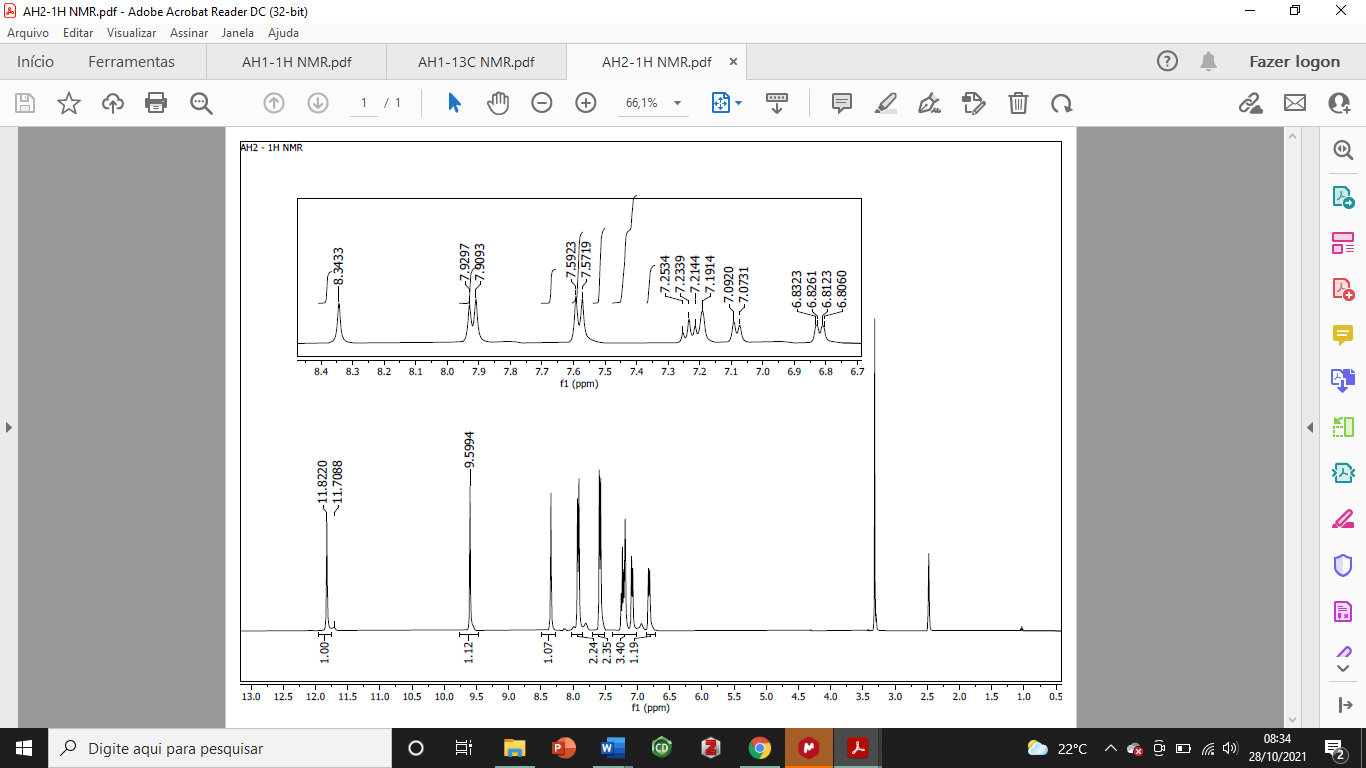


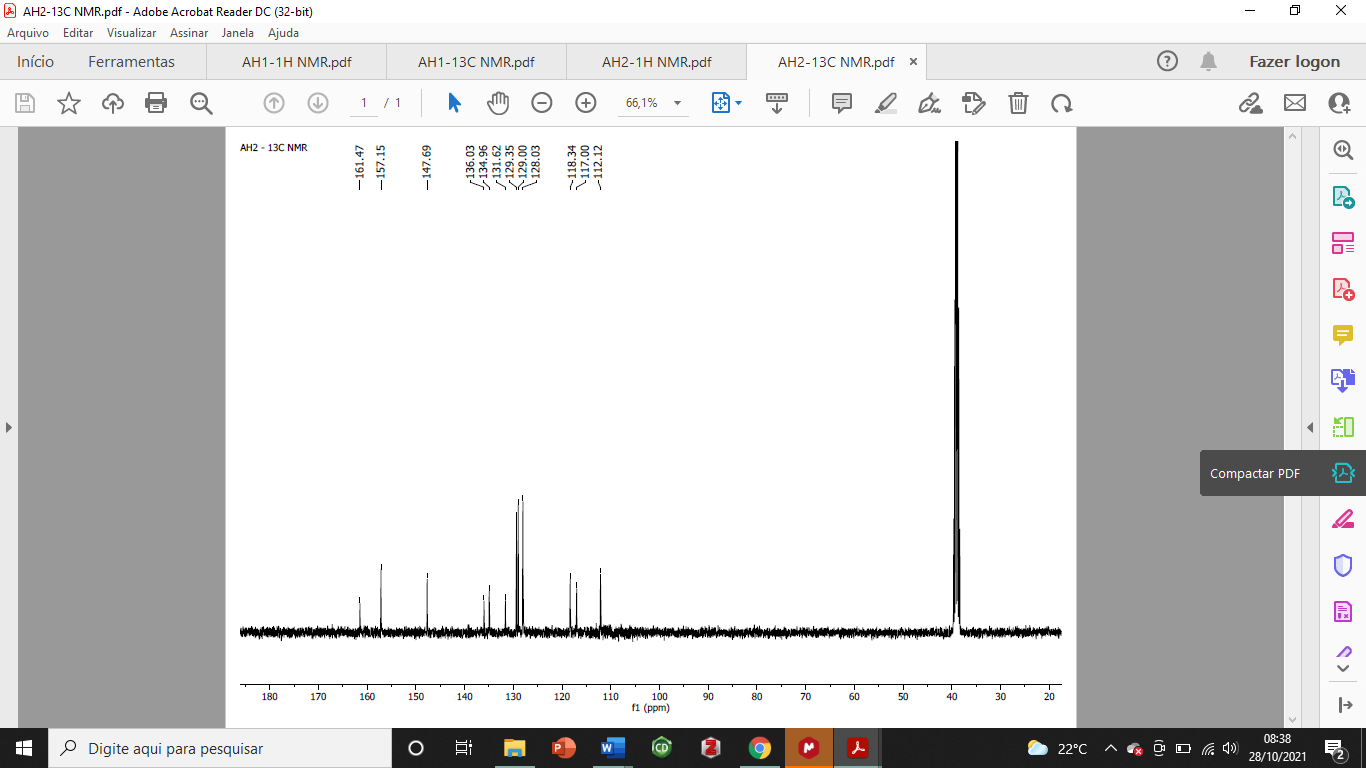


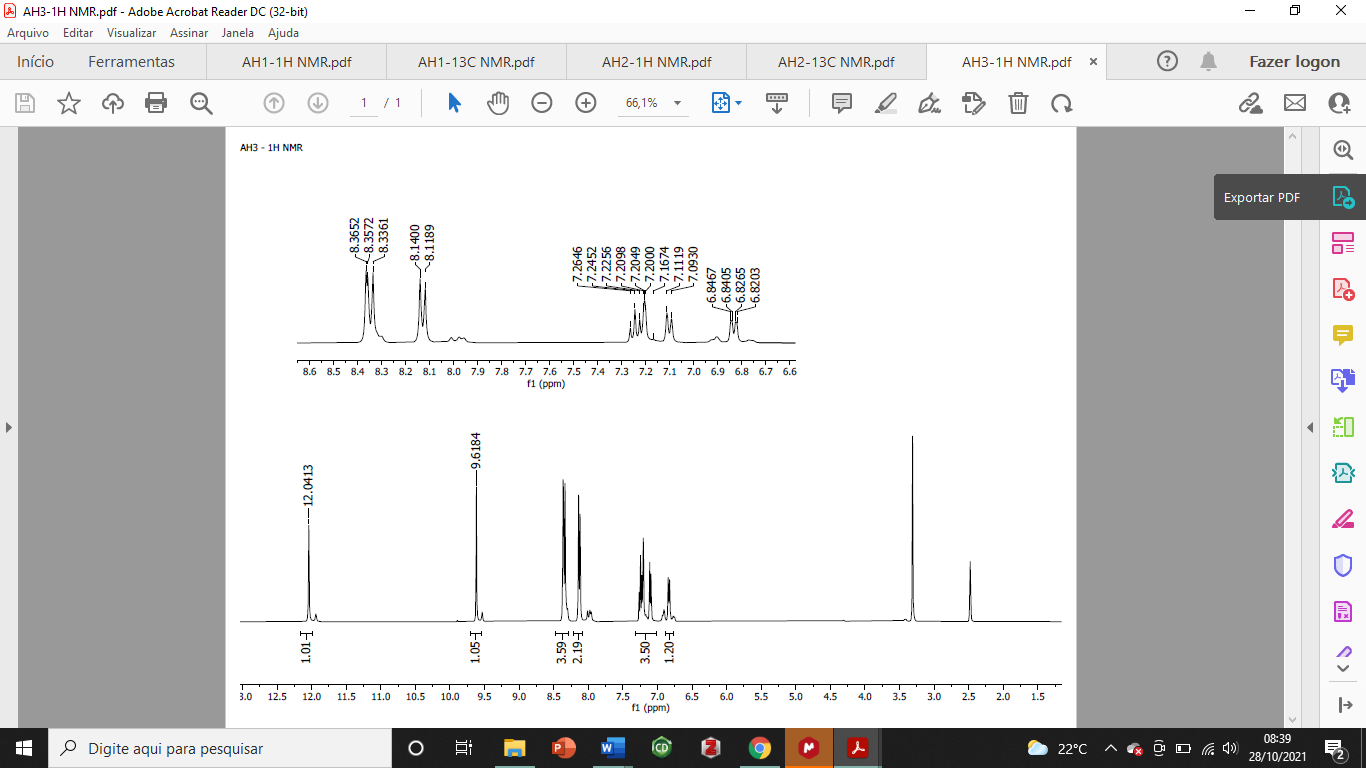


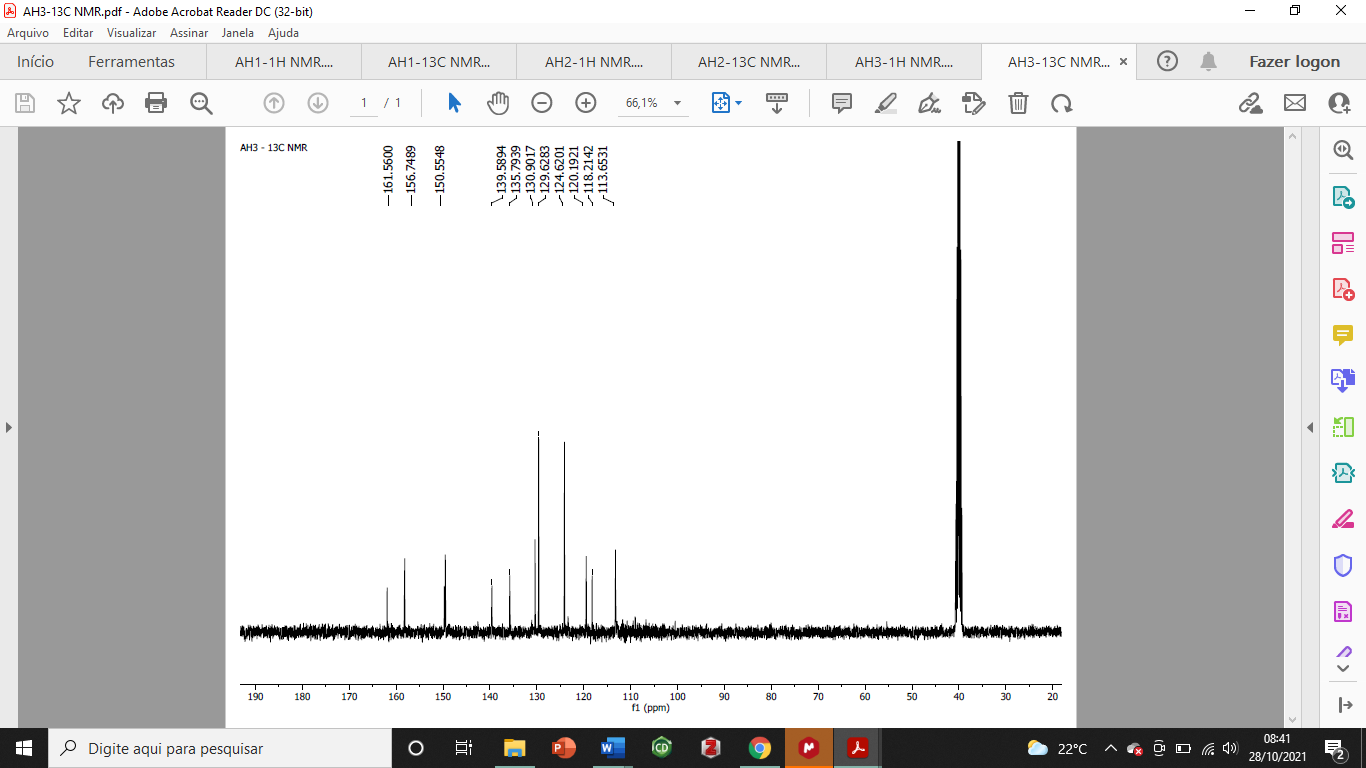


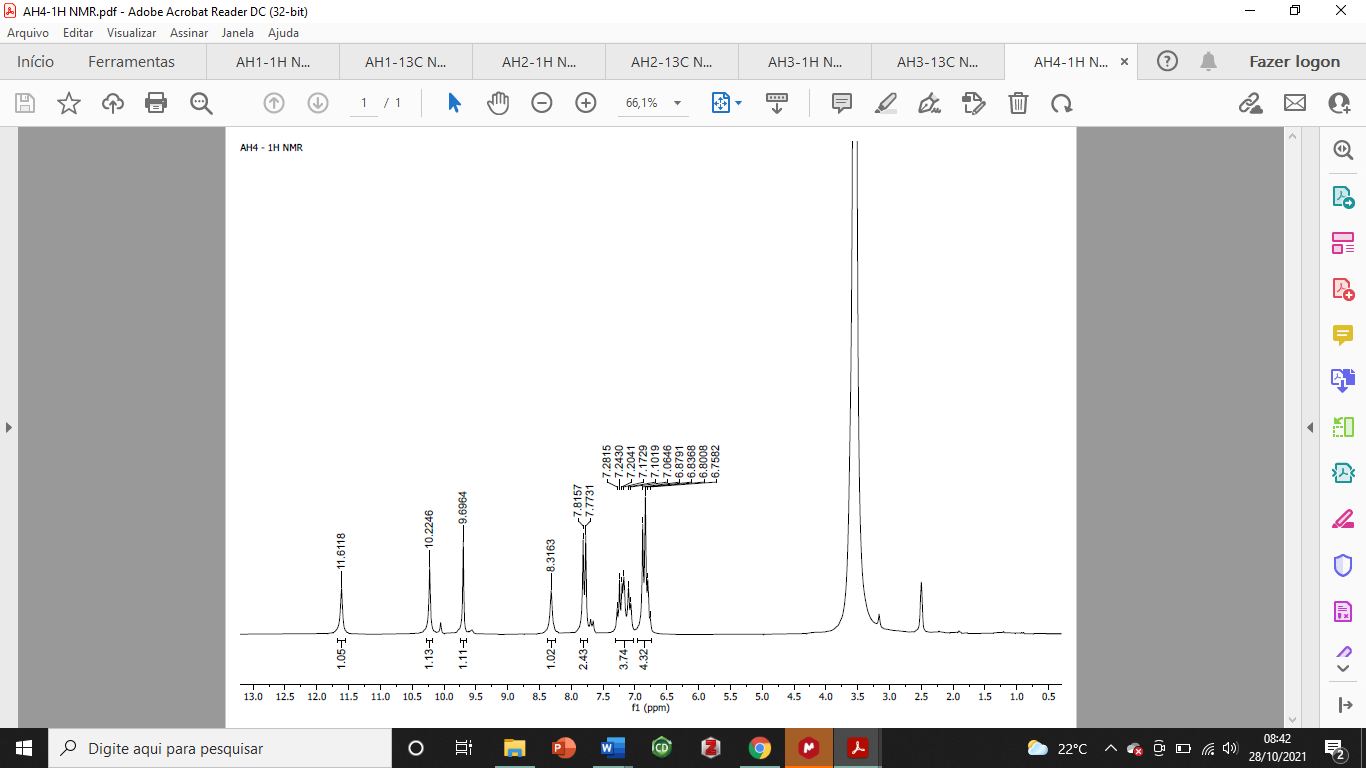


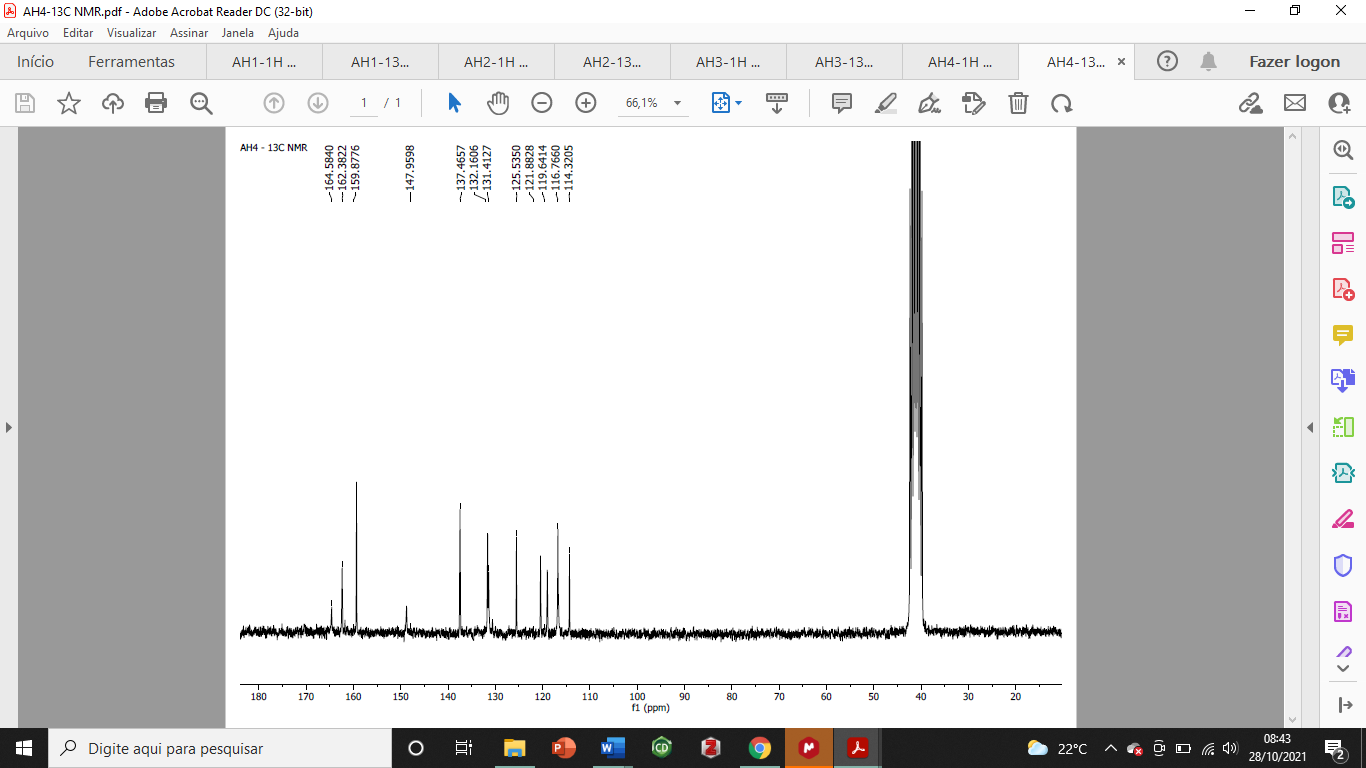


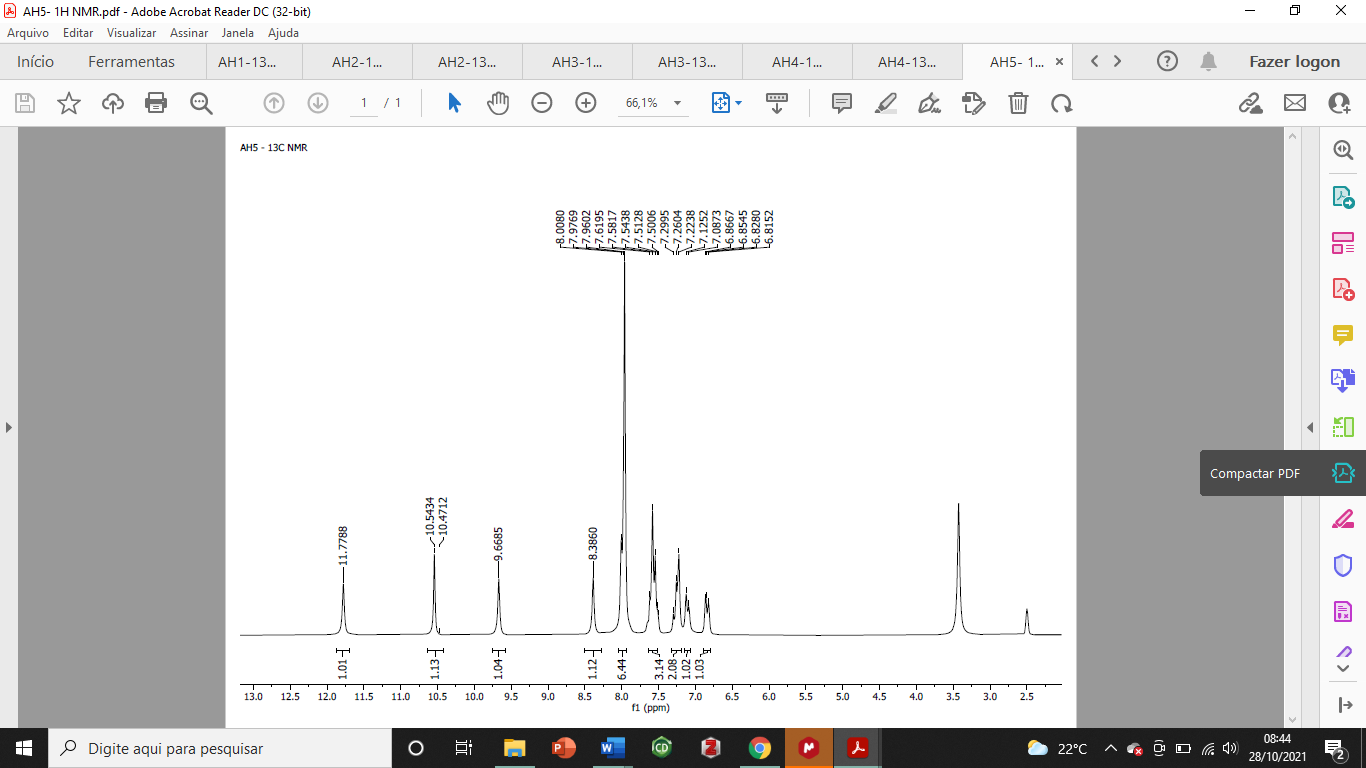


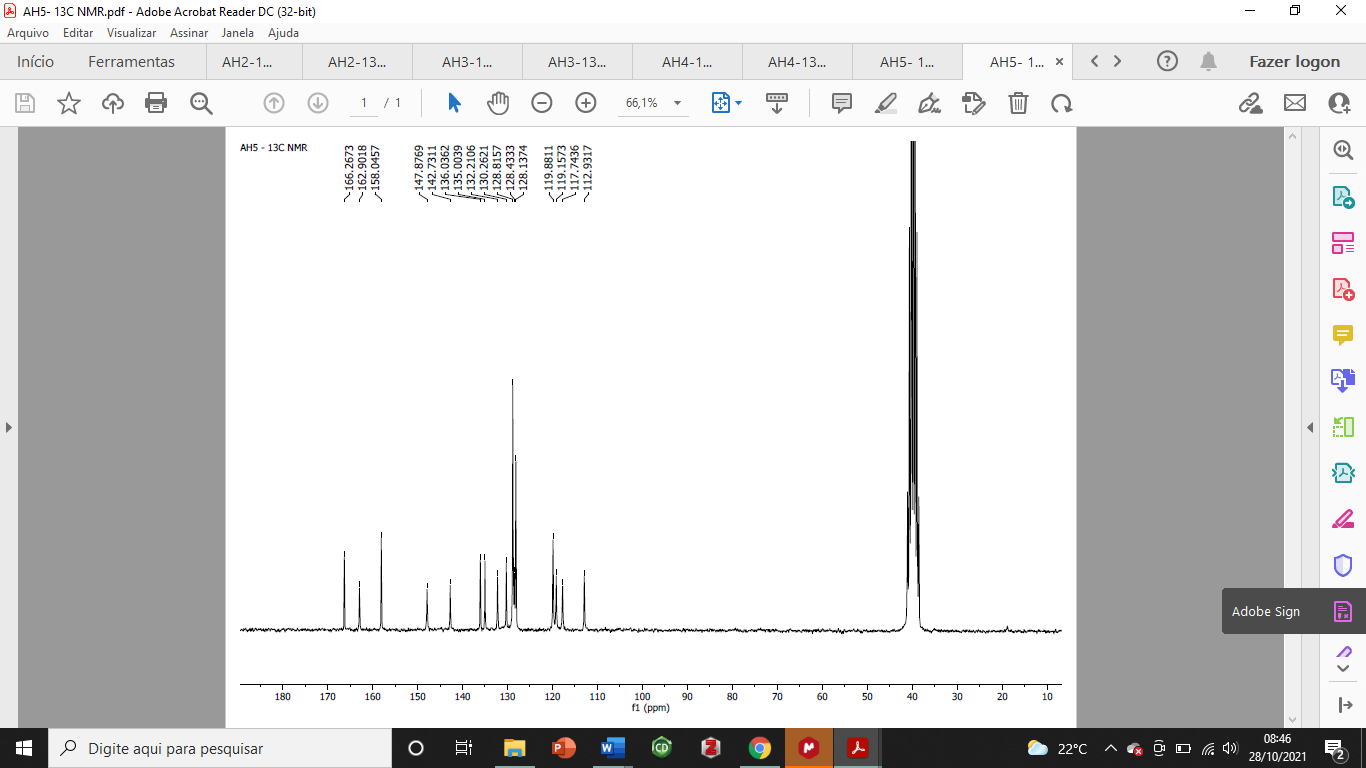


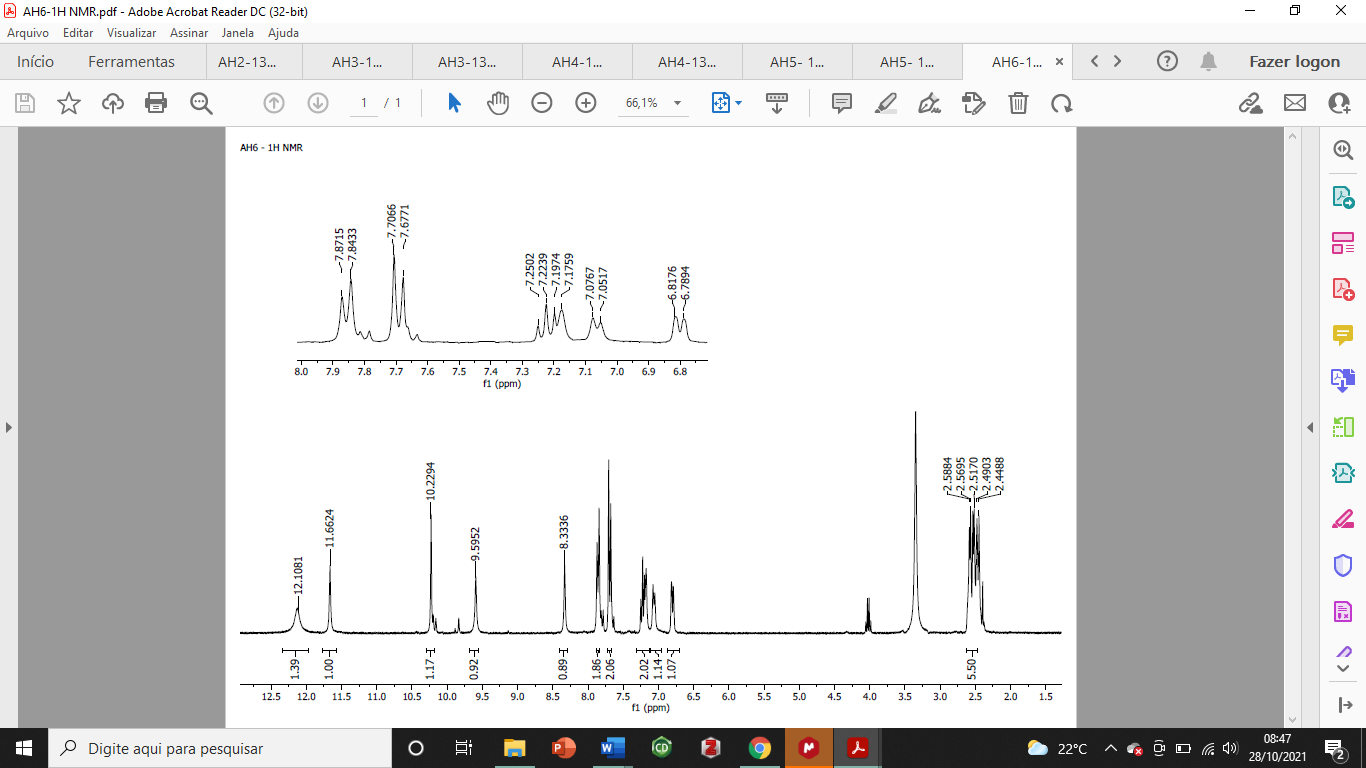


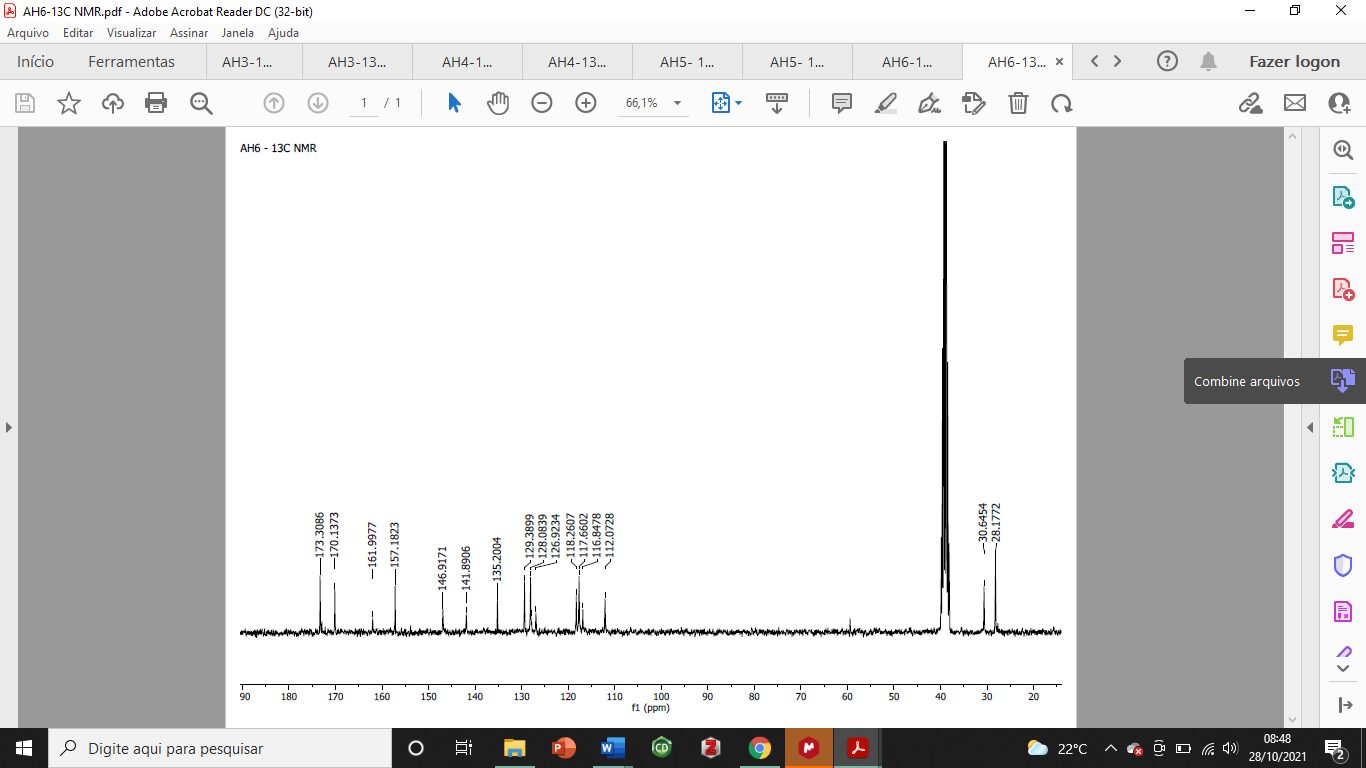


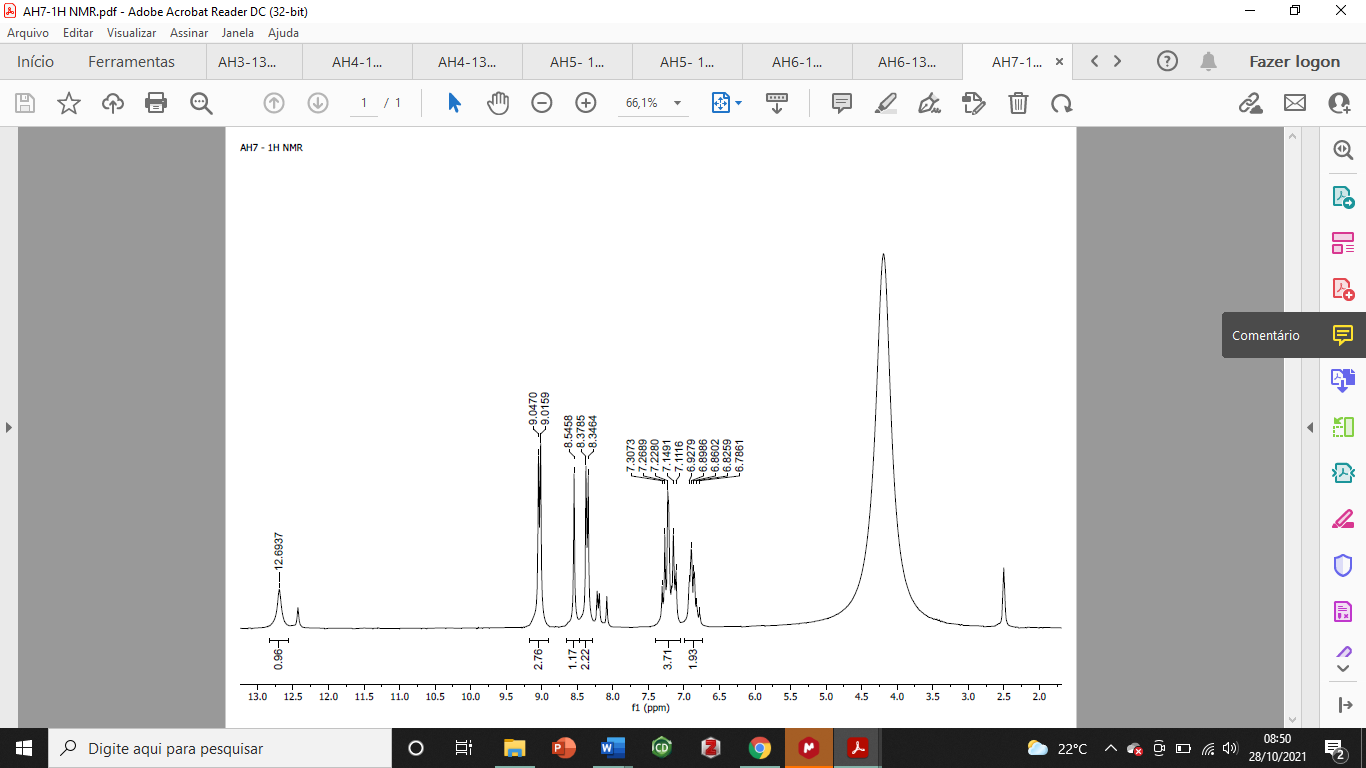


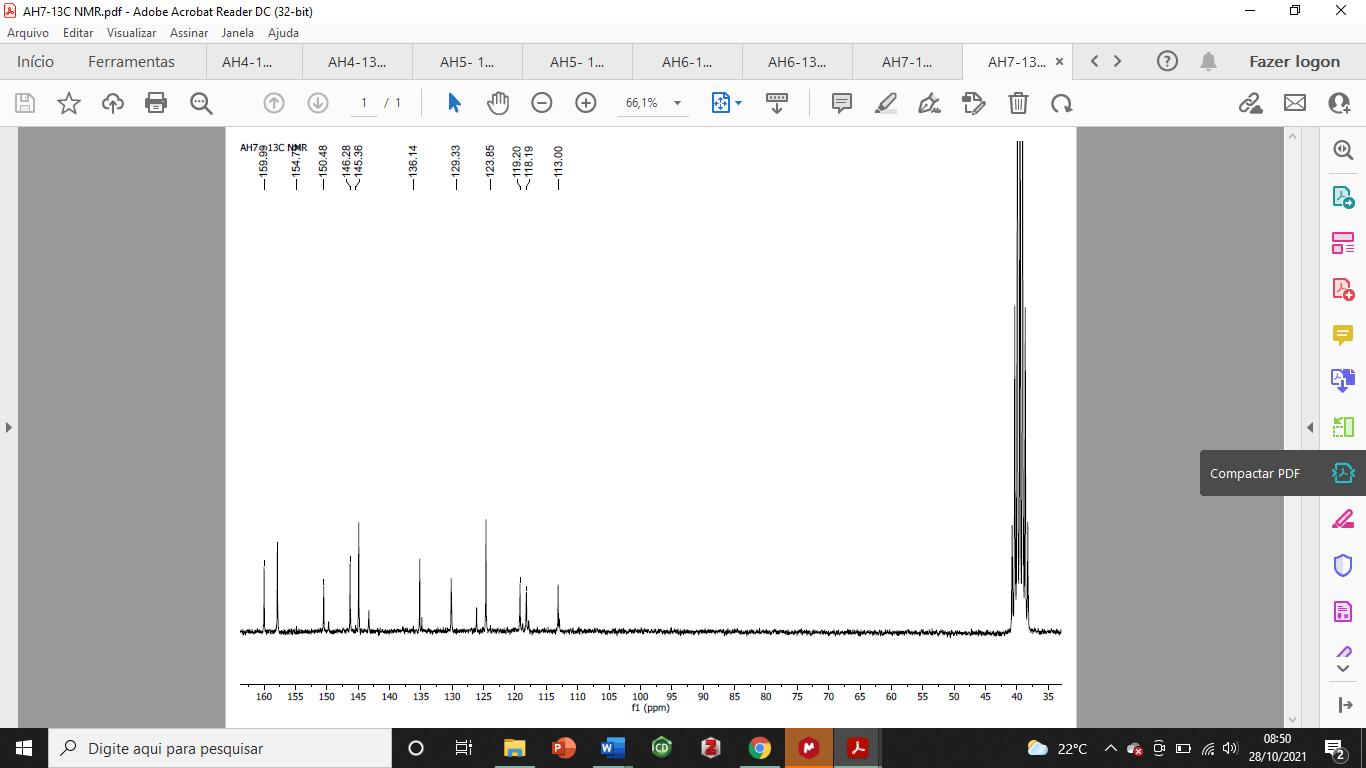


**Mass Spectrometry Spectra**


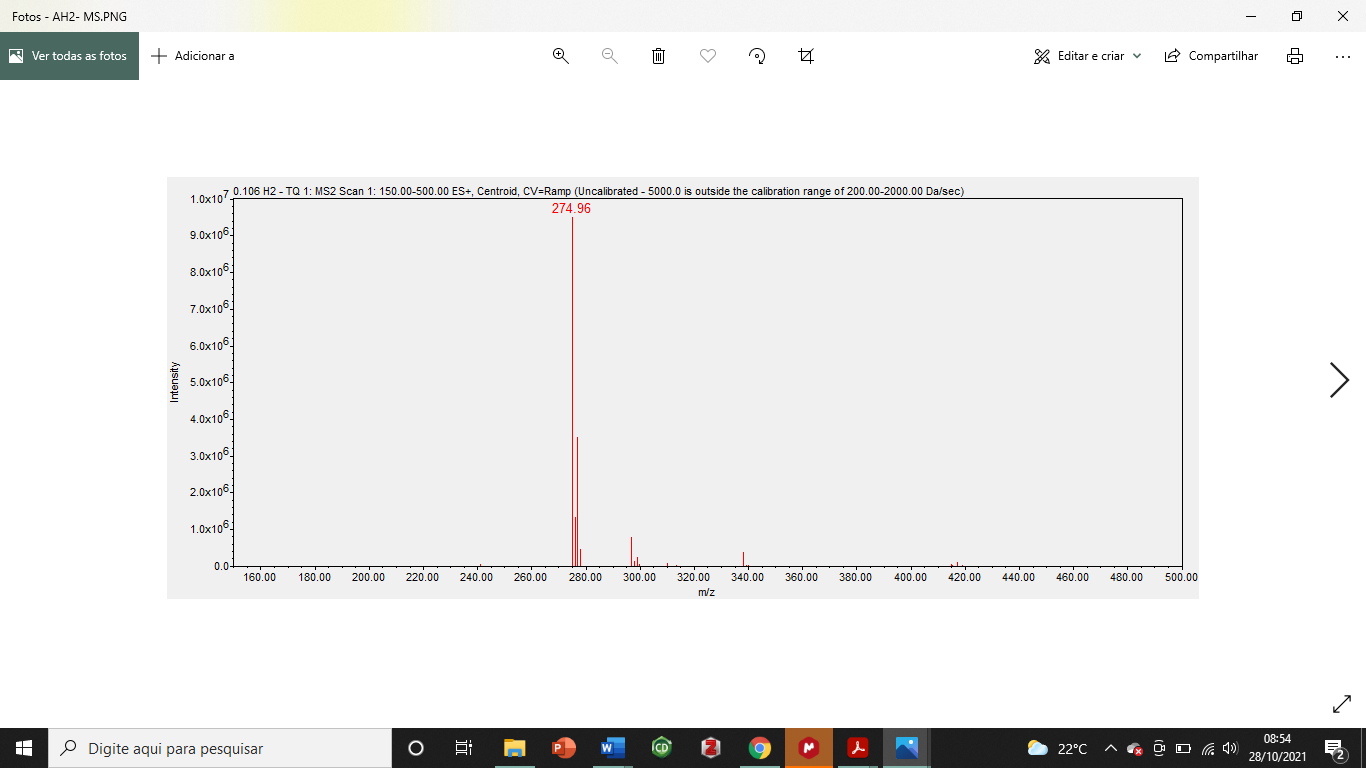


MS spectrum for AH2


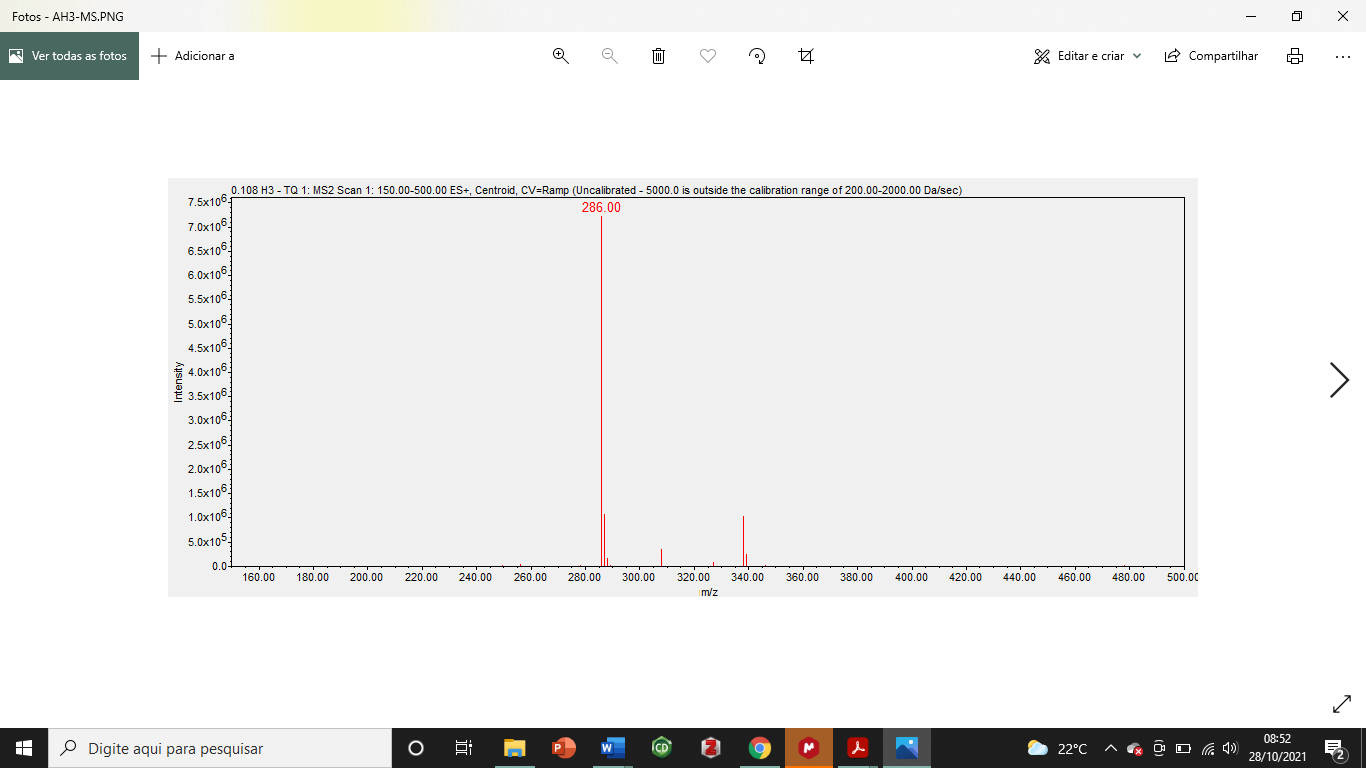


MS spectrum for AH3


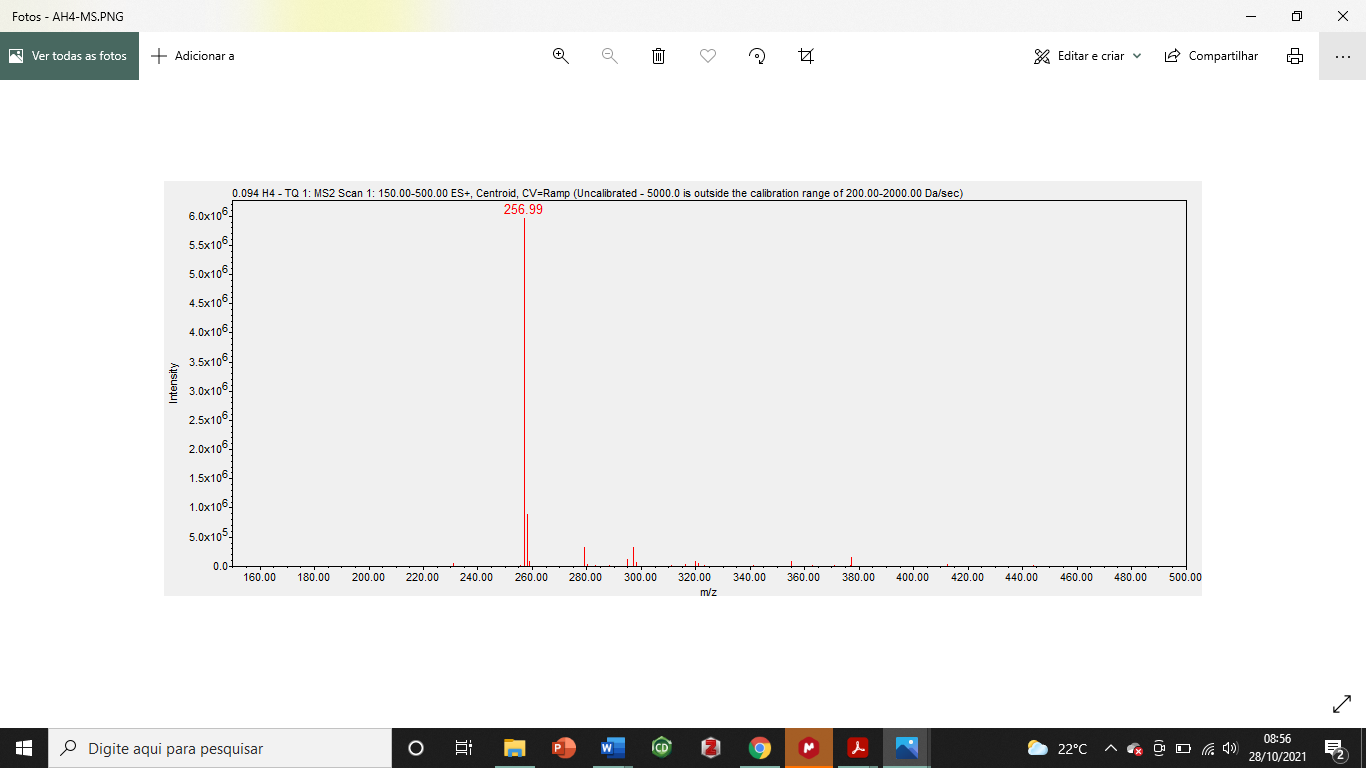


MS spectrum for AH4


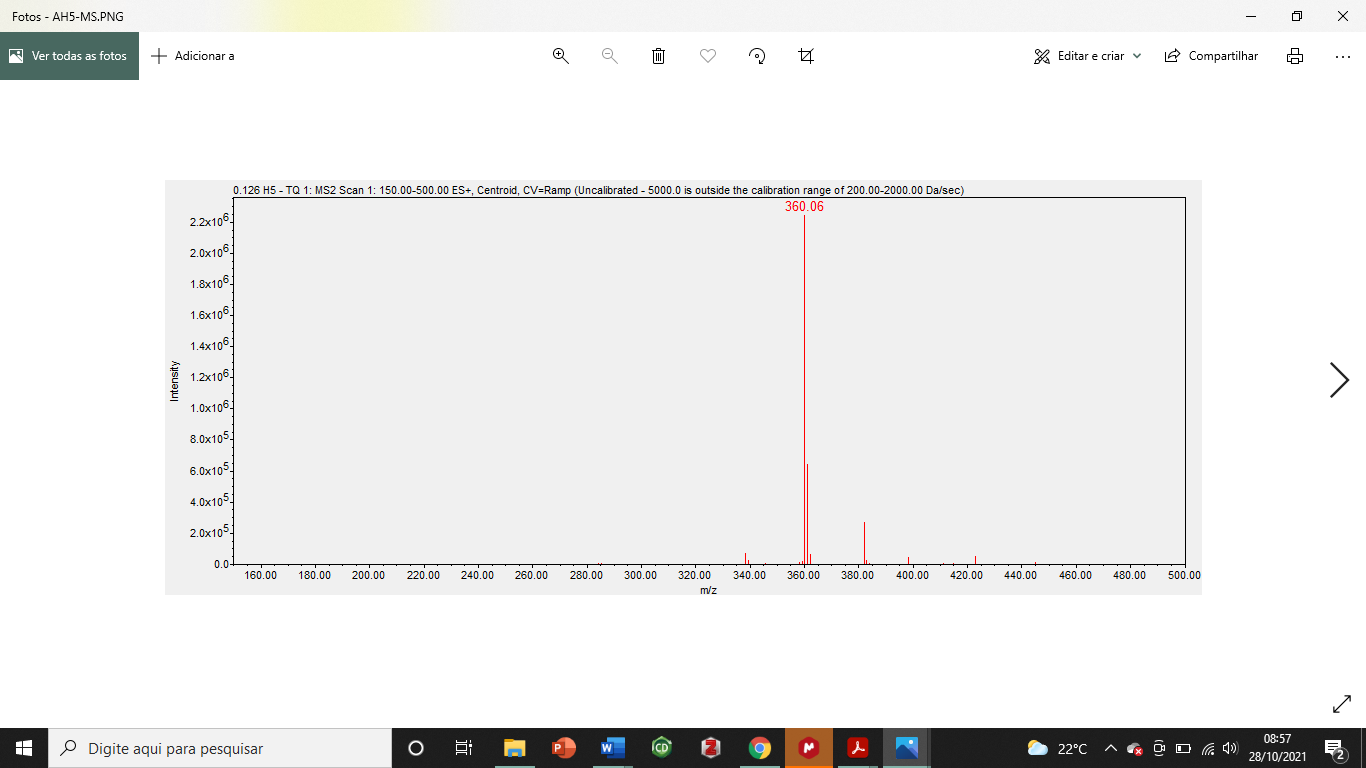


MS spectrum for AH5


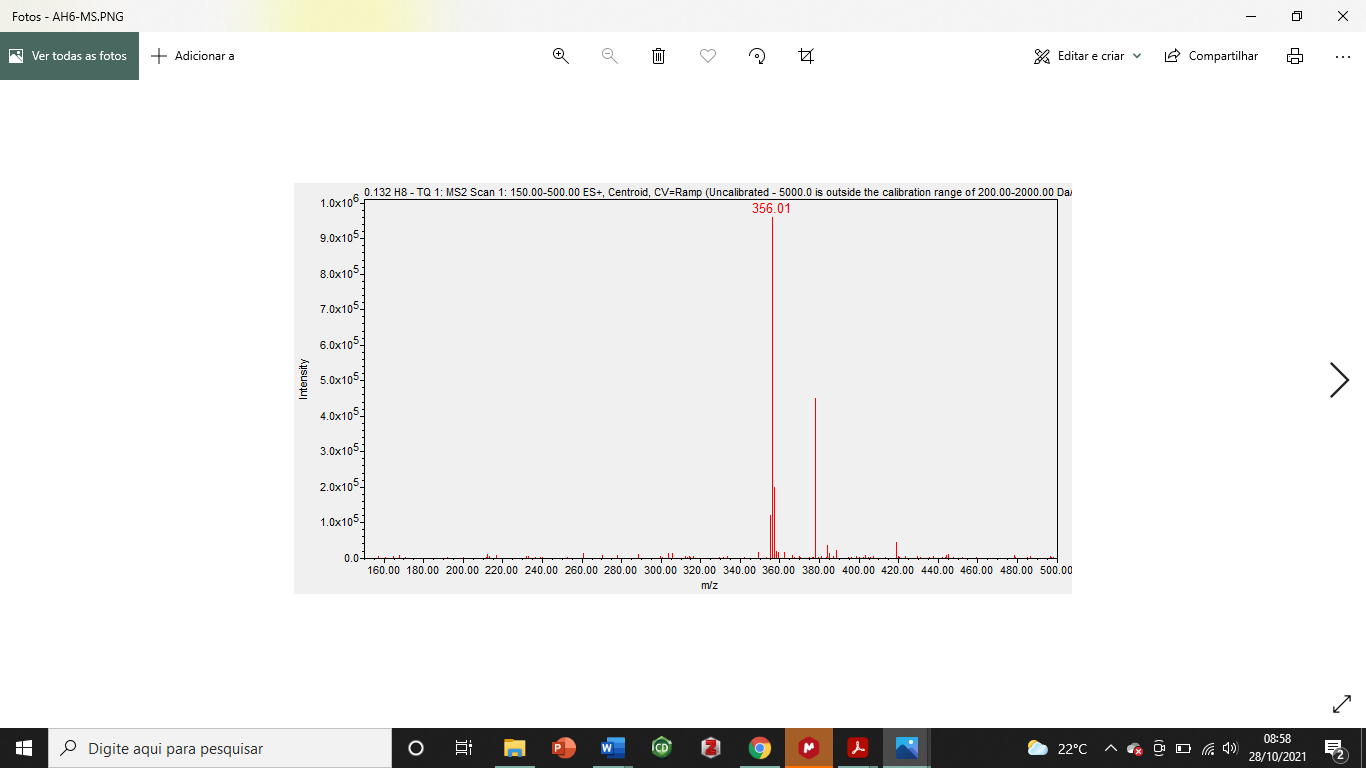


MS spectrum for AH6


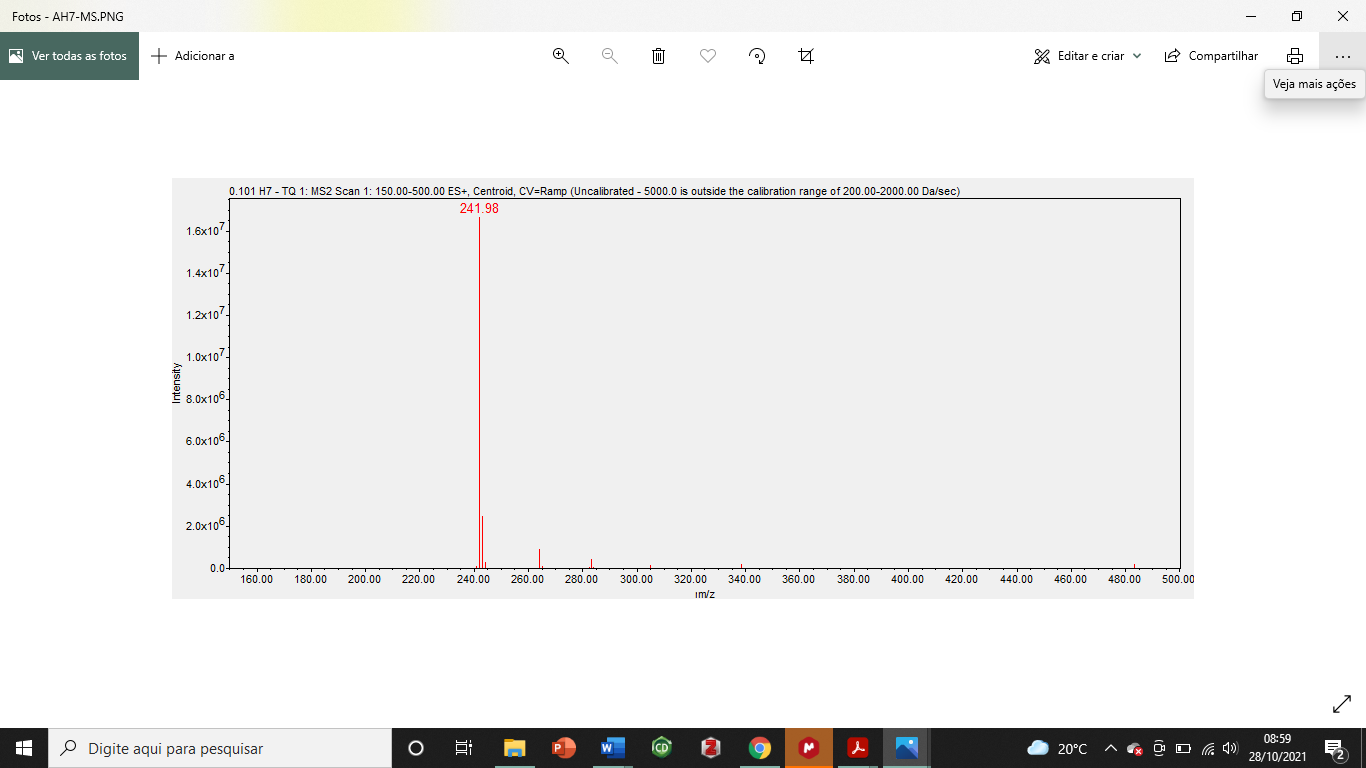


MS spectrum for AH7
